# Hepatitis B virus infection with high HBsAg and undetectable DNA levels in untreated blood donors: Viral mechanisms and transfusion safety implications

**DOI:** 10.1111/trf.70334

**Published:** 2026-07-20

**Authors:** Maelenn Païline Delcourt, Marion Vermeulen, Aneta Kopacz, Manuela Miletic, Andrew Saville, Christoph Niederhauser, Jean‐Michel Pawlotsky, Syria Laperche, Daniel Candotti

**Affiliations:** ^1^ Department of Blood Transmitted Agents National Institute of Blood Transfusion Paris France; ^2^ Institut Mondor‐(INSERM U955), Team “Viruses, Hepatology, Cancer” Créteil France; ^3^ Department of Virology Henri Mondor Hospital (AP‐HP), University of Paris‐Est Créteil France; ^4^ The South African National Blood Service Johannesburg Republic of South Africa; ^5^ The University of the Free State Bloemfontein Republic of South Africa; ^6^ Department of Virology Institute of Haematology & Transfusion Medicine Warsaw Poland; ^7^ Croatian Institute of Transfusion Medicine Zagreb Croatia; ^8^ Interregional Blood Transfusion SRC Berne Switzerland; ^9^ Institute for Infectious Diseases, University of Berne Berne Switzerland; ^10^ Etablissement Français du Sang La Plaine Saint‐Denis France

## Abstract

**Background:**

Blood donor screening occasionally identifies individuals with high HBV surface antigen (HBsAg) levels despite undetectable or extremely low HBV DNA. Molecular basis and transfusion risk of this discordant profile remain unclear.

**Study Design and Methods:**

Seventy‐five blood donors with HBsAg >100 IU/mL and undetectable or very low HBV DNA (<6 IU/mL), including 39 ID‐NAT‐nonreactive, were investigated. HBV DNA was further assessed using a second ID‐NAT assay, confirmatory PCR following virion concentration, and sequencing. Sequences were compared with those from genotype‐matched high‐viremia controls. Selected basic core promoter (BCP) and core mutations were introduced into 1.18‐mer replication‐competent Hepatitis B virus (HBV) plasmids to assess effects on extracellular virion‐associated DNA production.

**Results:**

Four (5%) samples were nonreactive by the two ID‐NAT assays. HBV DNA was confirmed in all donors, often at unquantifiable levels, and all were anti‐HBc positive. No consistent molecular signature was identified. Heterogeneous, case‐specific mutations were detected in BCP, core, and PreS1, including variants predicted to affect pregenomic RNA transcription, encapsidation, or virion assembly, with no evidence of impaired polymerase activity. In vitro, selected BCP mutations significantly reduced extracellular HBV DNA without affecting HBsAg production. Most core mutations had minimal impact, although two strains carrying cN92T/cM93T or cS170F showed reduced virion‐associated DNA. Some constructs exhibited impaired virion secretion or increased naked capsid production.

**Conclusion:**

HBsAg‐positive/low‐DNA profile largely reflects chronic inactive infection with minimal viremia. Diverse regulatory and structural mutations may contribute in a multifactorial manner. Persistent viral genomes and residual replication support continued HBsAg screening where NAT sensitivity is suboptimal and anti‐HBc testing cannot be implemented.

**Abbreviations**
AU: arbitrary unit
BCP: basic core promoter
DNA: deoxyribonucleic acid
HBc: hepatitis B core
HBeAg: hepatitis B e antigen
HBsAg: hepatitis B s antigen
HBV: hepatitis B virus
ID‐NAT: individual donation nucleic acid testing
LLOQ: lower limit of quantification
LoD: limit of detection
PCR: polymerase chain reaction
RCHP: recombinant replication‐competent HBV plasmids
SEAP: secreted human placental alkaline phosphatase

## 1 INTRODUCTION

Monitoring circulating HBsAg and HBV DNA provides complementary insights into HBV biology and disease progression, reflecting viral gene expression and replication, respectively. Although these markers often correlate during acute infection, this association frequently weakens during inactive chronic infection, particularly in asymptomatic blood donors.^1^, ^2^

Routine large‐scale screening of blood donors for HBsAg and HBV DNA ensures transfusion safety and provides insight into inactive chronic HBV infection in untreated populations.^3^ Screening assays used in blood donation are highly sensitive and differ from diagnostic assays used in symptomatic patients. They may reveal atypical profiles, including: (i) occult HBV infection (OBI), defined by very low HBV DNA in the absence of detectable HBsAg; and (ii) persistent HBsAg reactivity with repeatedly undetectable or barely detectable HBV DNA.^4^, ^5^ While OBI has been extensively studied, the biological basis, prevalence, and clinical implications of sustained HBsAg‐reactive/HBV DNA‐nonreactive infection in treatment‐naïve individuals remain poorly defined.

The perceived redundancy of testing three HBV markers (HBsAg, anti‐HBc, and HBV DNA) has led to debate about discontinuing HBsAg screening in settings using HBV NAT, particularly in low‐endemic regions where anti‐HBc testing is performed.^6^ Reported rates of HBsAg positivity with undetectable HBV DNA range from 1.0 to 70.3 per 100,000 donations, likely reflecting differences in endemicity, assay sensitivity, and confirmation algorithms.^5^, ^6^, ^7^ Clarifying the molecular basis of HBsAg reactivity in the absence of detectable HBV DNA is essential to assess infectious risk and the safety of discontinuing HBsAg testing. Excess HBsAg is mainly produced from HBV DNA integrated into hepatocyte DNA, whereas virions originate from HBV cccDNA. However, discordance may also result from variants with impaired genome replication, virion assembly, or secretion.^8^, ^9^

This study aimed to identify molecular mechanisms associated with sustained HBsAg production and extremely low HBV DNA levels in asymptomatic untreated individuals and to evaluate potential implications for transfusion safety.

## 2 MATERIALS AND METHODS

### 2.1 HBV blood screening and sample selection

The blood donations screening used ABBOTT PRISM HBsAg (95% limit of detection [LoD]: 0.022 IU/mL; Abbott Laboratories, Abbott Park, IL, USA), Architect HBsAg (LoD: 0.036 IU/mL; Abbott) or Monolisa HBsAg Ultra (LoD: 0.025 IU/mL; BioRad, Marnes‐la‐Coquette, France), and individual donation nucleic acid testing (ID‐NAT) with the Procleix‐Ultrio Plus or Procleix‐Ultrio Elite and HBV discriminatory assays (95% LoD: 4.1–4.5 IU/mL; Grifols, Barcelona, Spain), hereafter referred to as ID‐NAT1.

In HBV‐reactive donors, HBsAg was quantified using the Architect HBsAg QT assay (Abbott Diagnostics). HBV DNA was quantified using the Cobas TaqMan HBV assay (lower limit of quantification [LLOQ]: 6 IU/mL; LoD: 3 IU/mL; Roche Diagnostics, Meylan, France).

Seventy‐five archived HBV‐reactive donor samples collected between 2010 and 2015 were retrospectively selected from Croatia (*n* = 3), France (*n* = 37), Poland (*n* = 4), and South Africa (*n* = 31). Selection criteria included HBsAg levels >100 IU/mL, and either ID‐NAT1 nonreactivity (*n* = 39) or ID‐NAT1 reactivity with HBV DNA load <6 IU/mL (*n* = 36) (Figure 1). All 75 case samples were anti‐HBc positive, and 42 were anti‐HBe positive, including nine with undetectable HBV DNA. None were HBeAg or anti‐HBs reactive (Table 1). A control group of 138 donors from similar origins with HBsAg >100 IU/mL and HBV DNA >10,000 IU/mL was included. The complete characteristics of the 75 donors and 138 controls are shown in Table 1.

**FIGURE 1:**
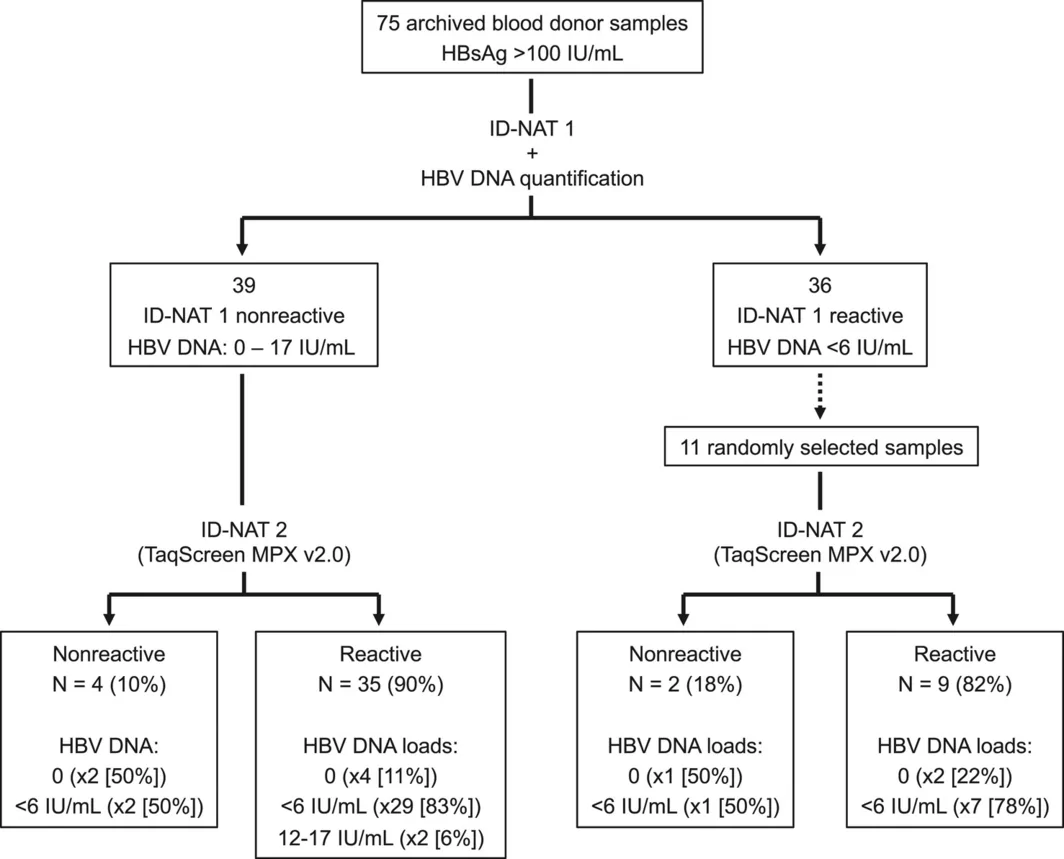
Blood donor sample selection and classification. ID‐NAT1: Procleix Ultrio Plus or Elite assays; ID‐NAT2: Cobas TaqScreen MPX Test v2.0. ID‐NAT, individual donation nucleic acid testing. HBsAg, hepatitis B surface antigen; HBV, hepatitis B virus.

**TABLE 1:** Characteristics of selected HBV‐infected individuals with HBsAg >100 IU/mL and undetectable or low viral DNA levels.

Parameters	Studied cases			Controls
	ID‐NAT1^a^ nonreactive	ID‐NAT1^a^ reactive	Total	
*N*	39	36	75	138
HBV DNA load				
Undetectable	6 (15.4%)	3 (8.3%)	9 (12.0%)	‐
Detectable (IU/mL) (median [range])	33 (84.6%) (<6 [<6–17])	33 (91.7%) (<6 [NA])	66 (88.0%) (<6 [<6–17])	138 (100%) (1.3 × 10^5^ [10^4^–10^9^])
Age (years)^b^ (median [range])	30 (18–60)	29 (18–61)	30 (18–61)	27 (18–67)
Gender^b^				
Male (%)	17 (45%)	22 (65%)	39 (54%)	108 (79%)
Female (%)	21 (55%)	12 (35%)	33 (46%)	29 (21%)
HBsAg (IU/mL) (median [range])	2.5 × 10^2^ (1.1 × 10^2^–9.4 × 10^3^)	2.8 × 10^3^ (1.5 × 10^2^–2.6 × 10^4^)	6.1 × 10^2^ (1.1 × 10^2^–2.6 × 10^4^)	3.6 × 10^4^ (5.4 × 10^2^–1.1 × 10^6^)
Anti‐HBc reactive (%)	39 (100%)	36 (100%)	75 (100%)	135 (98%)^c^
Anti‐HBe reactive/tested^d^ (%)	7/7 (100%)	34/35 (97%)	41/42 (98%)	NT (‐)
HBV genotypes (%)^e^				
A1	15 (38.5%)	3 (8.3%)	18 (24%)	15 (11%)
A2	4 (10.3%)	7 (19.4%)	11 (15%)	13 (9.5%)
A3	‐	2 (5.6%)	2 (3%)	‐
B	‐	2 (5.6%)	2 (3%)	14 (10%)
C	1 (2.55%)	‐	1 (1%)	13 (9.5%)
D	5 (12.8%)	9 (25%)	14 (19%)	44 (32%)
E	1 (2.55%)	8 (22.2%)	9 (12%)	39 (28%)
D/E		1 (2.8%)	1 (1%)	‐
Undetermined	2 (5.1%)	1 (2.8%)	3 (4%)	‐
NA	11 (28.2%)	3 (8.3%)	14 (18%)	‐

*Note*: HBV_A1_ is overrepresented due to South African samples.

Abbreviations: HBsAg, hepatitis B surface antigen; HBV, hepatitis B virus; ID‐NAT, individual donation nucleic acid testing; NA, no applicable; NT, not tested.

^a^ ID‐NAT1: initial HBV DNA screening performed with the Procleix‐Ultrio Plus or Elite assay.

^b^ Data missing for three cases.

^c^ Three anti‐HBc nonreactive samples confirmed to be early acute HBV infection.

^d^ 42 of 75 samples were tested.

^e^ Undetermined, only BCP/PC sequence not suitable for genotyping; D/E, recombinant genotype.

### 2.2 HBV complementary testing

HBsAg, anti‐HBc, anti‐HBs, and HBeAg/anti‐HBe were further tested using Murex HBsAg v3 (DiaSorin, Dartford, UK), Monolisa Anti‐HBc PLUS, Monolisa Anti‐HBs PLUS, and Monolisa HBe Ag‐Ab PLUS (Bio‐Rad, Marnes‐la‐Coquette, France), respectively.

HBV DNA was subsequently tested using the Cobas TaqScreen MPX Test v2.0 (95% LoD: 2.3 IU/mL; Roche Diagnostics, Mannheim, Germany), hereafter referred to as ID‐NAT2. In addition, viral particles were concentrated from 5 to 12 mL of plasma by ultracentrifugation.^10^ Nucleic acids were then extracted using the High Pure Viral Nucleic Acid Kit. Nearly full‐length HBV genomes (3160 bp) and specific subgenomic regions (Precore/Core, Pre‐S/S, S, and basic core promoter/precore [BCP/PC]) were amplified by nested PCR and sequenced.^10^, ^11^

HBV RNA was quantified in plasma after reverse transcription using specific real‐time PCR in a one‐step procedure, as previously described.^12^ HBV RNA levels were quantified against a standard curve of 10‐fold serially diluted synthetic HBV RNA and expressed as arbitrary units (AU).

### 2.3 HBV sequence analyses

A phylogenetic analysis was performed in MEGA‐X (neighbor‐joining, Kimura two‐parameter distance model, 1000 bootstrap replicates) with GenBank HBV genotype A–H reference sequences. Nucleotide and deduced amino acid sequences were compared with genotype‐matched consensus sequences from HBsAg+/HBV DNA+ donors.

### 2.4 Cell culture transfection with 1.18‐mer replication‐competent HBV DNA plasmids

Recombinant replication‐competent HBV DNA plasmids (RCHPs) were generated by synthesizing 1.18‐mer HBV sequences from samples and controls of genotypes A–E (Genecust, Boynes, France), and cloning them into pBluescript II SK+ vector. Site‐directed mutagenesis was performed using the QuikChange II Site‐directed Mutagenesis kit (Agilent Technologies, Cedar Creek, TX, USA).

RCHPs were transfected into HuH‐7 cells (500 ng/50,000 cells) using FuGENE HD (Promega, Madison, WI, USA). Transfection efficiency was monitored by co‐transfecting the pSELECT‐zeo‐SEAP plasmid (50 ng/50,000 cells) and measuring human placental alkaline phosphatase (SEAP) activity (InvivoGen, San Diego, CA, USA). Each construct was tested in at least two independent duplicate experiments.

Six days post‐transfection, the culture supernatants and cells were collected. HBsAg levels in extracellular and intracellular fractions were measured and normalized to SEAP activity as previously described.^13^

### 2.5 Extracellular production of HBV DNA

Clarified supernatants (500 μL) were treated with 12% PEG‐8000 overnight at 4°C to precipitate viral particles. The particles were then pelleted by centrifugation, resuspended in water, and treated with DNase.^10^ Capsid‐protected HBV DNA was extracted and quantified by in‐house qPCR (95% LoD: 20 IU/mL).^11^ Residual plasmid DNA was assessed by qPCR targeting vector sequences (pSK‐F 5′‐GCCGGAAGCATAAAGTGTAAAGC‐3′ and pSK‐R 5′‐GCTGGCACGACAGGTTTC‐3′; probe pSK‐MGB 5′‐[FAM]‐TTGCGCTCACTGCCC‐[MGBEQ]3′). The fold changes of extracellular HBV DNA for mutant versus wild‐type RCHPs were calculated using the 2^−ΔΔCt^ method.^14^ Viral particles were analyzed by native agarose electrophoresis and Southern blot hybridization using a biotin‐labeled full‐length HBV probe.^15^

### 2.6 Statistical analysis

Categorical and continuous variables were analyzed using Fisher's exact and Mann–Whitney tests. A *p*‐value <0.05 was considered significant.

## 3 RESULTS

### 3.1 Sample characteristics

As shown in Table 1, HBsAg differed significantly between ID‐NAT1 nonreactive (median: 2.5 × 10^2^ IU/mL; range: 110–9.4 × 10^3^) and ID‐NAT1 reactive cases (median: 2.8 × 10^3^ IU/mL; range: 150–2.6 × 10^4^) (*p* < .0001), as well as between cases and controls (median: 3.6 × 10^4^ IU/mL; range: 536–1.1 × 106) (*p* < .0001).

Among ID‐NAT1 nonreactive samples, 35/39 (90%) were HBV DNA‐reactive by ID‐NAT2 assay (Figure S1). This included four samples for which the HBV DNA level could not be quantified (Figure 1). Four (10%) remained nonreactive in both ID‐NAT assays, including two with HBV DNA between the LoD and the LLOQ and two undetectable. HBV DNA was confirmed in these four after viral particle concentration. Among 11 randomly selected ID‐NAT1 reactive samples, two were ID‐NAT2 nonreactive. One sample had an undetectable HBV DNA and an HBsAg level of 2011 IU/mL, while the other had an HBV DNA level <6 IU/mL and an HBsAg level of 1536 IU/mL. Ultimately, HBV DNA was confirmed in all 75 samples using four detection methods (Table S1). Nine (12%) had detectable but non‐quantifiable DNA, and 66 (88%) had quantifiable DNA (6–17 IU/mL) (Figure 1 and Table 1).

### 3.2 HBV sequencing and genotype determination

HBV DNA was amplified by nested PCR after viral particle concentration. Nearly full‐length genome, Pre‐S/S, and BCP/PC regions were amplified in 31/75 (41%), 53/75 (71%), and 48/75 (64%) samples, respectively. Additional amplification of S and PreCore/Core regions in previously unamplified samples yielded 4/22 (18%) and 2/22 (9%) amplicons, respectively. Overall, HBV DNA was confirmed by sequencing in 61/75 samples (81%). Full‐length genome or Pre‐S/S sequences were generated in 26/39 (67%) of ID‐NAT1 nonreactive and 32/36 (89%) of reactive samples.

Phylogenetic analyses and genotyping were conducted using full‐length, Pre‐S/S, PreCore/Core, and S sequences. HBV control genotypes were previously determined from Pre‐S/S sequencing. Genotypes A–E were identified in both groups (Table 1). Case sequences were aligned with 255 genotype‐matched, full‐length genomes from HBsAg‐positive/HBV DNA‐positive (>10^3^ IU/mL) donors, including 18 HBV_A1_ (South Africa), 41 HBV_A2_ and 53 HBV_D_ (Europe), 37 HBV_B_ and 44 HBV_C_ (Southeast Asia), and 62 HBV_E_ (West Africa).

### 3.3 Sequence analysis of the BCP/PC region

BCP/PC sequences from 49 case strains (16 HBV_A1_, 10 HBV_A2_, 2 HBV_A3_, 2 HBV_B_, 1 HBV_C_, 11 HBV_D_, and 7 HBV_E_) were analyzed (Figure S2). Strain B6538 exhibited an 8‐nucleotide deletion between pseudo‐TATA boxes TA2 and TA3 (nt 1763–1770) compared to the HBV_A2_ reference sequence (GenBank accession number KY003230). Additional mutations disrupting the PreCore ORF included a one‐nucleotide deletion (nt1839) in strain SA5256, a one‐nucleotide insertion (nt1840) in strains B5168 and B6536, and a two‐nucleotide insertion (nt1851) in strain B5672. A two‐nucleotide insertion (nt1821–1822) was observed in the pregenomic RNA (pgRNA) lnr element of strain B5594. Twelve strains carried substitutions in the 5′ proximal pgRNA ε stem‐loop, some predicted to alter RNA secondary structure or reverse transcription priming (e.g., G1862U/G1888U [strain SA2882], C1877U/G1887A [strain SA9624], C1860A/G1899A [strain B5594], and G1888U [strain SA5699]).

### 3.4 Analysis of the HBV core protein

Full‐length core sequences were available for 35 case strains (10 HBV_A1_, 9 HBV_A2_, 1 HBV_C_, 10 HBV_D_, and 5 HBV_E_). The average intra‐group amino acid variability was higher in the HBV_A1_, HBV_A2_, and HBV_E_ cases than in the controls (3.3% vs. 0.5%, 8.2% vs. 6.2%, and 7.4% vs. 2.2%; *p* < .0001), but lower in HBV_D_ cases (5.1% vs. 7.1%; *p* < .0001). Seven case strains (4 HBV_A1_, 2 HBV_A2_, and 1 HBV_E_) had sequences identical to their genotype‐matched consensus. Amino acid substitutions occurred in the N‐terminal (aa1–140) and/or C‐terminal (CTD; aa150–185) domains, including sites previously linked to capsid assembly, virion production, and replication, with additional uncharacterized substitutions (Figure S3).

Several case‐specific mutations absent in controls may impair core protein structure, including cP45S (strain PO2270), cP50H/A (strains B5168, B5129, B5545, PO1125, and B6197), cP79Q (strains SA018, B7112, and B7169), cE113Q (strains SA7504, B5175, B5672, B6197, B6536, B7052, and B7181), cA131P (strains SA018, SA5409, SA5699, SA7504, B6197, and B7052), and cP135L/Q/T (strains B6197, B5654, B5503, and B5594). Four HBV_A1–2_ case strains (strains SA2882, SA5699, B5654, and B5672) harbored unique cD153N/Y/Q changes within the genotype A‐specific DR insertion of the first poly‐arginine CTD domain, while HBV_C_ strain B5129 showed an RR insertion at the same site. Additional unique changes included deletions at cE77 (strains B7181 and B6197) and cT147 (strain PO2896), a premature stop at c184 (strain SA5699), and a 372‐nt deletion extending the C‐terminus by 54 aa (strain B5558).

### 3.5 Analysis of Pol proteins and of levels of HBV‐RNA

Complete Pol sequences were obtained for 29 cases (Figure S6). Amino acid variability across the four functional Pol domains was similarly limited in cases and controls, except in genotype E, where case sequences showed significantly higher variability in the terminal protein and spacer domains (Figure 2 and Table S3). Motifs essential for HBV polymerase activity were conserved, including Y65 and AGILYKR (156–162) in the terminal protein, catalytic residues D431, D553, and D554 in the reverse transcriptase domain, and D450, D702, E731, and D790 in the RNase H domain (Figure S6). Plasma HBV‐RNA levels were assessed in 37 available cases (including four with undetectable viral load) and 25 controls as an indirect marker of impaired DNA synthesis. HBV‐RNA could not be detected in 33/37 cases and in 9/25 controls. Extremely low levels (<1 AU/mL) were detected in 4/37 cases and 3/25 controls, and higher levels of HBV‐RNA were detected in 13/25 controls, not in the 37 cases (median: 310; range: 30–5600 AU/mL) (Figure S7).

**FIGURE 2:**
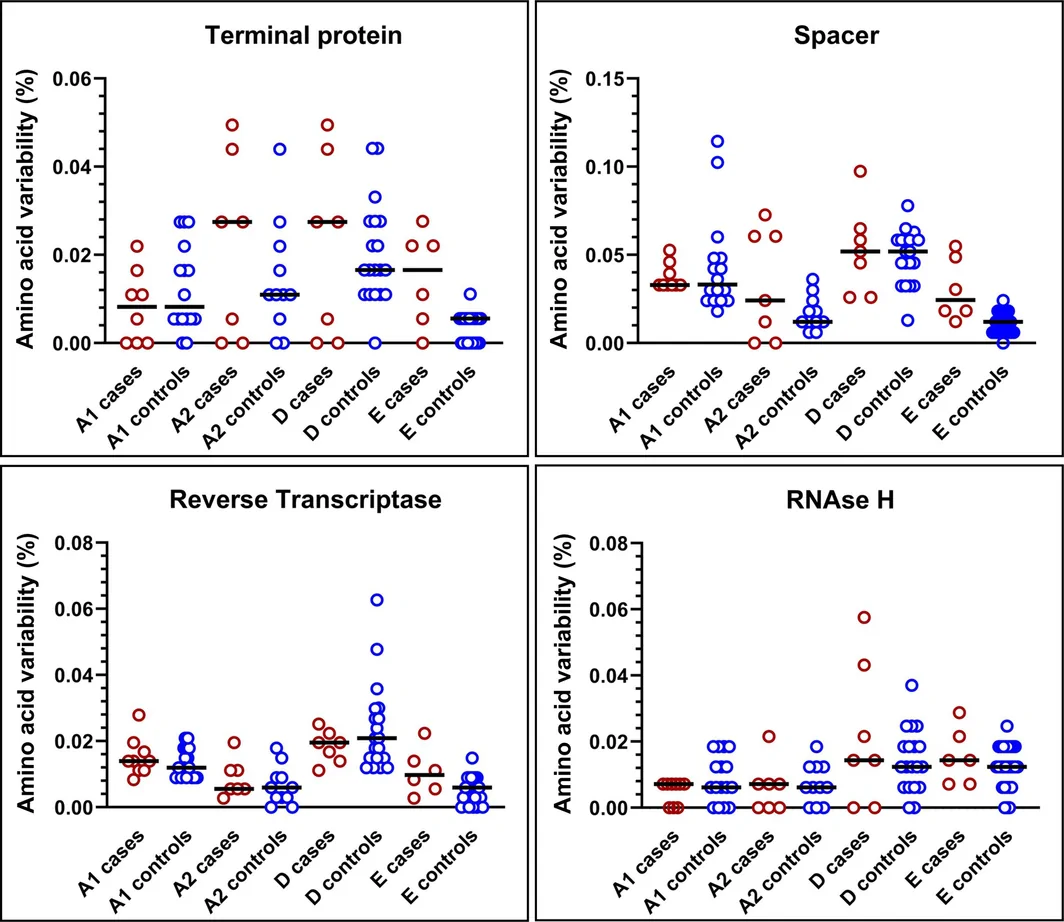
Amino acid diversity distribution in cases and controls across Pol domains. Amino acid sequences from cases and controls are represented by red (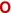) and blue (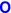) circles, respectively. Pol domains are indicated.

### 3.6 Sequence analysis of HBV surface proteins

Large S sequences were obtained in 50 cases (Figures S4 and S5). Overall aa variability was low but higher than in the controls for genotypes A2 (3.0% [0.0–4.9] vs. 2.0% [0.0–6.5]), D (3.6% [1.4–6.5] vs. 2.5% [0.0–7. 9]) and E (3.6% [1.3–6.0] vs. 1.0% [0.0–4.5]) (*p* < .0001), with no difference in A1 (2.8% [0.0–4.1] vs. 3.3% [0.3–7.0]). PreS1 showed an average mutation rate of 2.7% (0–10 substitutions), highest in the HBV_C_ strain B5129 (10/119 aa). One to three substitutions in the putative core‐binding domain were detected in 12 strains, including 10 case‐specific changes (e.g., Q104K [strain B5168], D114E [strain B5654], S115T [strain B5558], S109T + Q118H [strain B7227], A118V + Q120R [strain B7152], M119T [strain B6078], M120R [strain SA4546], and M118I + Q119R [strain B6197]), and an arginine insertion at position 101 (strain PO1125). Strain B6078 lack the PreS1 and PreS2 start codons.

### 3.7 In vitro HBV virion production

Replication‐competent 1.18‐mer HBV plasmids were generated from six control strains (genotypes A1‐E) and five case strains (pSKB5129, pSKB5168, pSKB5503, pSKB6156, and pSKPO1125) with available full‐length genome sequences carrying case‐specific BCP and/or core mutations (Table 2). After transfection into HuH‐7 cells, total HBsAg levels were comparable to controls for pSKB5168 and pSKB6156 but significantly reduced for pSKB5129, pSKB5503, and pSKPO1125 (Figure 3A). pSKPO1125 also showed marked intracellular HBsAg retention (Figure 3B).

**TABLE 2:** Replication‐compatible HBV DNA plasmids (replication‐competent HBV DNA plasmids [RCHPs]).

Plasmids	Status	HBV genotype	Mutations of interest	
			BCP	Core
pSKSA9241	Control	A1		
pSKB8131	Control	A2		
pSKM90	Control	B		
p3.8II	Control	C		
pSKP127	Control	D		
pSKB6037L87	Control	E		
pSKB5129	ID‐NAT1 nonreactive	C	T1815G, A1818G	cP130Q, cR151P, c153NR insertion
pSKB5168	ID‐NAT1 reactive	A2	T1840 insertion	
pSKB5503	ID‐NAT1 reactive	D		cP135T
pSKB6156	ID‐NAT1 reactive	E		cS168F
pSKPO1125	ID‐NAT1 reactive	D		cD29H, cP50H
pSKSA9241‐del63	SDM	A1	8‐nt deletion 1763–1770	
pSKSA9241‐T77A87	SDM	A1	C1877T, G1887A	
pSKSA9241‐92H	SDM	A1		cN92H
pSKSA9241‐131P	SDM	A1		cA131P
pSKB8131‐TT52	SDM	A2	TT1852 insertion	
pSKB8131‐50H	SDM	A2		cP50H
pSKB8131‐92TT	SDM	A2		cN92T + cM93T
pSKB8131‐113Q	SDM	A2		cE113Q
pSKB8131‐130A	SDM	A2		cP130A
pSKB8131‐170F	SDM	A2		cS170F
pSKP127‐AC21	SDM	D	A1821/C1822 insertion	
pSKP127‐A60A99	SDM	D	C1860A, G1899A	
pSKP127‐del77	SDM	D		cE77 deletion
pSKP127‐79Q	SDM	D		cP79Q
pSKP127‐155 T	SDM	D		cS155T

*Note*: SDM: Case mutations introduced in RCHP controls by site directed mutagenesis.

**FIGURE 3:**
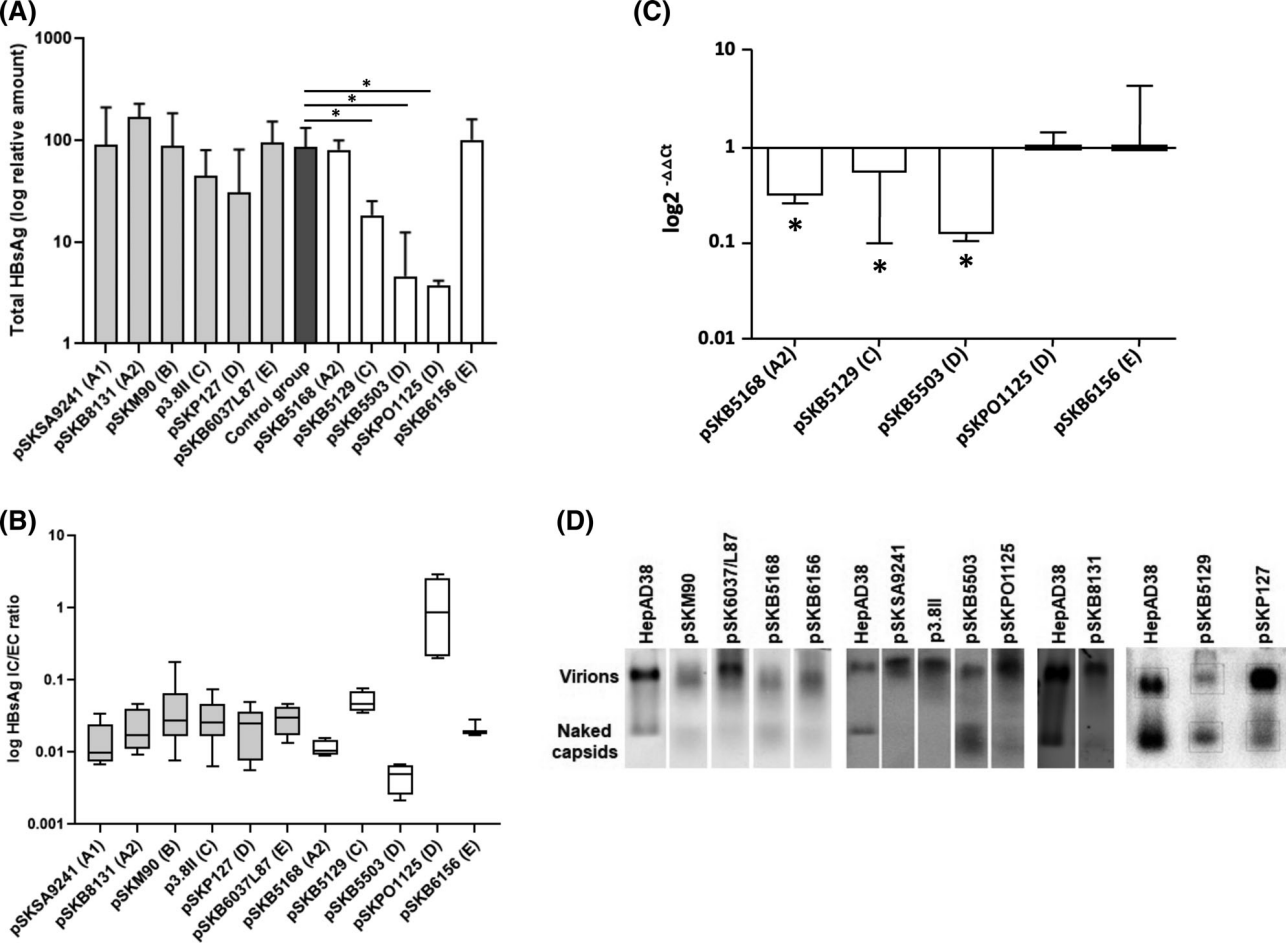
Analysis of extracellular hepatitis B surface antigen (HBsAg) and virion production in Huh‐7 cells transfected with recombinant, replication‐competent hepatitis B virus DNA plasmids. (A) Average individual extracellular HBsAg production in HuH‐7 cells transfected with five HBV variants (white bars) and six controls (gray bars); the black bar represents the mean control value; standard deviations are shown for three or more independent cultures, and HBV genotypes are indicated in parentheses. (B) The HBsAg intracellular/extracellular ratio observed in the variants (white bars) and the controls (gray bars). (C) Mean relative levels of extracellular HBV DNA production from the studied HBV variants expressed as a log value proportional to the DNA levels obtained from genotype‐matched controls. (D) Distribution of HBV DNA associated with virions and naked capsids; viral DNA was detected by Southern blot after particle segregation by electrophoresis in a native agarose gel. *, statistical significance reached (*p* <0.05).

Extracellular HBV DNA was quantified in culture supernatants after PEG precipitation, DNase I digestion, and normalization to residual plasmid DNA. Controls showed similar levels (data not shown). Case plasmids pSKB5168, pSKB5129, and pSKB5503 had significantly reduced extracellular HBV DNA, whereas pSKPO1125 and pSKB6156 were similar to controls (Figure 3C). Southern blotting confirmed virion‐associated HBV DNA in all samples, with generally low levels of naked capsid DNA (Figure 3D). Notably, pSKB5129 and pSKB5503 produced relatively more naked capsids than complete virions.

### 3.8 In vitro functional analysis of BPC and core mutations

Five case‐derived BCP mutations (deletion nt1763‐70, AC insertion nt1821, TT insertion nt1852, C1860A/G1899A, and C1877T/G1887A) were introduced into genotype‐matched RCHP controls (Table 2). HBsAg production was largely unaffected, except for a slight decrease with the nt1763‐70 deletion. In contrast, all mutations reduced extracellular HBV DNA (Figure 4). The nt1763‐70 deletion and the AC insertion at nt1821 also significantly decreased pgRNA levels in transfected cells (Figure 5).

**FIGURE 4:**
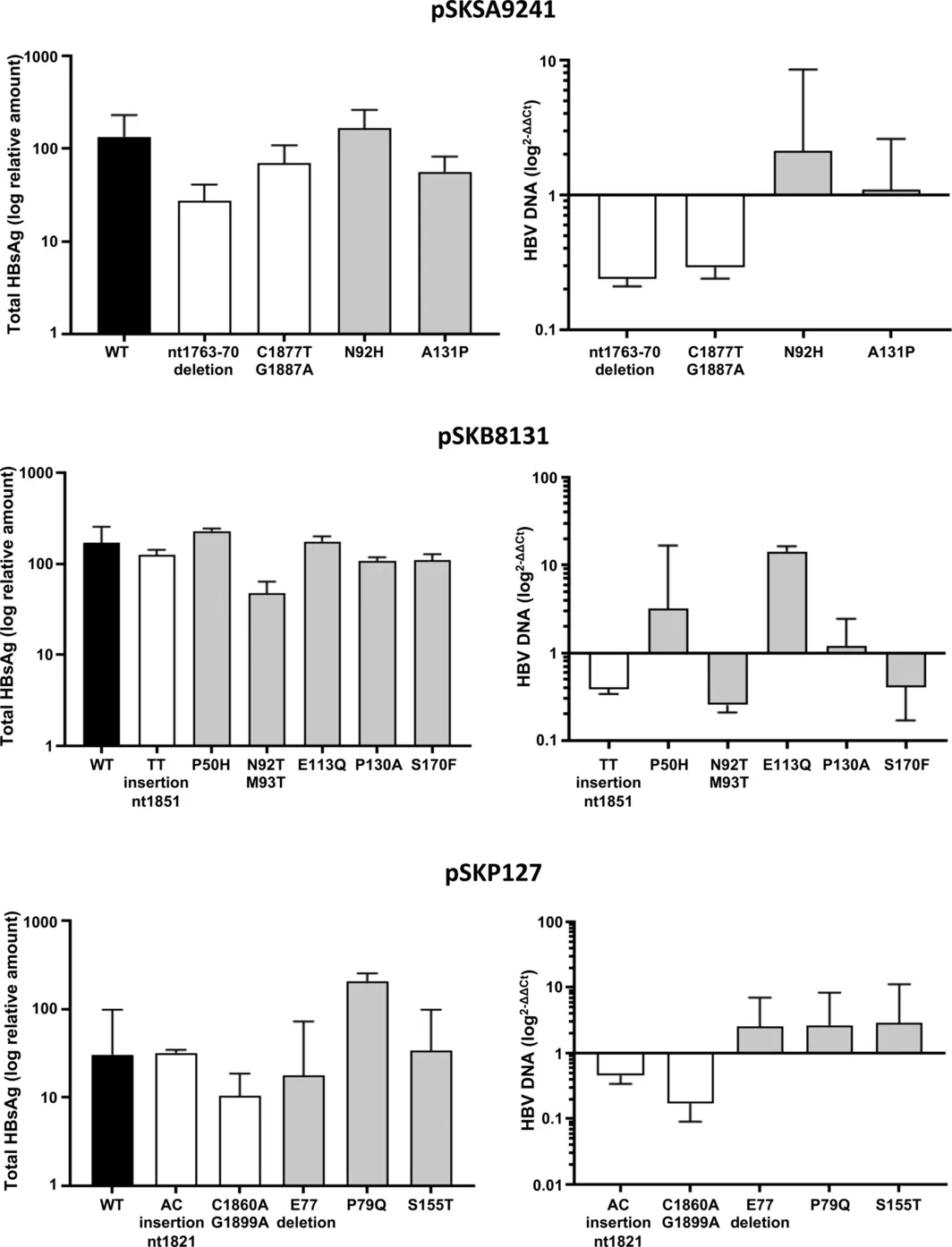
Impact of basic core promoter (BCP) and core mutations on the extracellular production of hepatitis B surface antigen (HBsAg) and hepatitis B virus DNA in transfected HuH‐7 cells. BCP mutations (white bars) and core mutations (gray bars) were introduced in genotype‐matched RCHP controls (HBV_A1_ pSKSA9241, HBV_A2_ pSKB8131, and HBV_D_ pSKP127). Normalized HBsAg levels (mean + standard deviation) are presented on the left, with the black bar representing the HBsAg production of the controls. The mean relative levels of extracellular HBV DNA production are expressed as a log value proportional to the DNA levels obtained from the controls.

**FIGURE 5:**
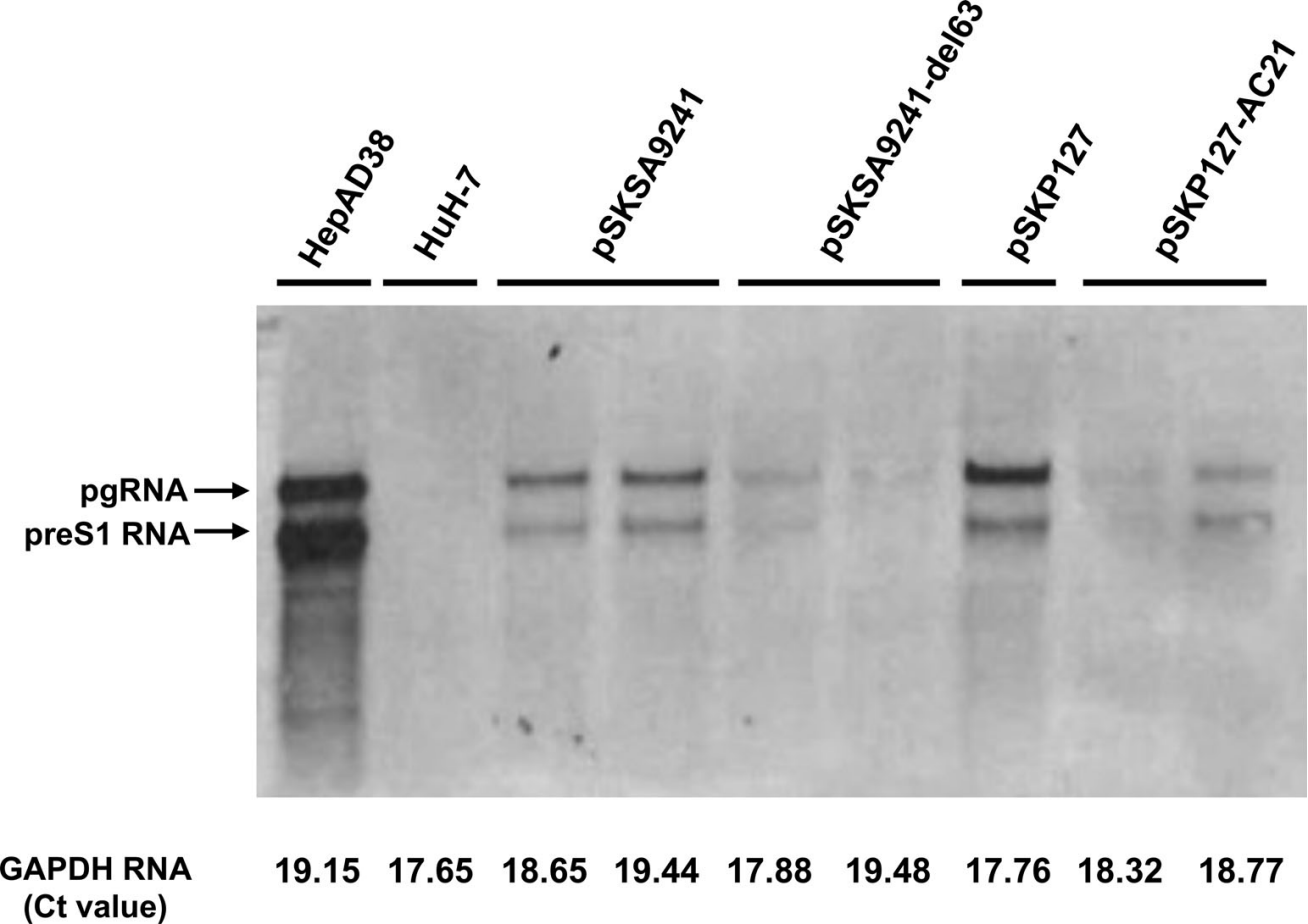
Impact of nt1763‐70 deletion and AC nt1821 insertion on hepatitis B virus pre‐genomic RNA production. The corresponding glyceraldehyde 3‐phosphate dehydrogenase. RNA quantification results obtained by quantitative reverse transcription polymerase chain reaction (Ct values) are shown as an internal control.

Ten case‐specific core mutations, absent in controls and potentially affecting nucleocapsid and/or virion formation, were introduced into genotype‐matched RCHPs (Table 2). Only cN92T/cM93T and cS170F reduced extracellular HBV DNA (Figure 4).

## 4 DISCUSSION

Screening of blood donors for HBV has identified asymptomatic carriers with high HBsAg levels despite undetectable or very low levels of HBV DNA in plasma. The viral mechanisms underlying this imbalance and the associated infectious risk remain unclear. This study selected 75 donor samples with HBsAg levels >100 IU/mL and undetectable or very low HBV DNA levels to investigate these viral mechanisms. These criteria were chosen to maximize phenotypic contrast and help identify differences in HBV replicative properties.

Several factors may account for the differences observed in HBsAg and HBV DNA levels in untreated individuals, including the large excess of subviral particles over infectious virions (10^4^–10^6^‐fold).^2^, ^16^, ^17^ False‐positive HBsAg reactivity was excluded using quantitative and neutralization assays (data not shown). HBV DNA detection by multiple molecular methods sometimes conflicted, mainly due to extremely low (<6–17 IU/mL) or undetectable levels, underscoring the need for highly sensitive assays. Notably, 70% of samples initially nonreactive by ID‐NAT1 were HBV DNA‐reactive by ID‐NAT2, consistent with the slightly higher sensitivity reported for ID‐NAT2 (4.1–4.5 vs. 2.3 IU/mL).^18^ NAT performance relies on the primer and probe set design. The number and genomic targets of the HBV primers and probes used in ID‐NAT1 and ID‐NAT2 assays are not publicly available. Limited reports suggest that the ID‐NAT2 assay targets the HBV core region.^19^, ^20^ However, no core‐region sequence data were available for ID‐NAT2 nonreactive samples. Although viral variants could potentially account for the lack of detection, particularly in the two samples with HBV DNA levels of 12 and 17 IU/mL, as well as for the discrepancies between assays, this remains speculative given the lack of sequencing data and the limited information available on the ID‐NAT1 target regions. Both assays missed 4 HBsAg‐positive samples (5.3%) detectable only after viral concentration or below the LLOQ (<6 IU/mL). All samples were anti‐HBc reactive, and 98% of the 42 tested were anti‐HBe reactive, regardless of HBV DNA. This HBsAg‐positive/anti‐HBc‐positive/anti‐HBe‐positive/HBV DNA‐low profile, observed across all genotypes, likely reflects chronic inactive infection with limited viral replication and low disease progression risk.^21^

Screening blood donors for HBV is essential for transfusion safety. HBsAg testing has long been the cornerstone of HBV screening, but the US Food and Drug Administration recently recommended its discontinuation due to HBV NAT implementation and potential anti‐HBc screening.^22^ However, it is unclear whether this can be widely implemented without compromising blood safety, as rare ID‐NAT nonreactive infections can still be transfusion‐transmitted.^2^, ^23^, ^24^, ^25^ Anti‐HBc testing combined with sensitive NAT could detect active and past infections, but in high‐endemic regions, it may unnecessarily exclude donors, reduce blood supply, and require complex confirmatory testing. Rare anti‐HBc‐negative donors with intermittent viremia, including one transfusion‐transmission case, have been reported.^26^, ^27^ NAT implementation is also costly and logistically challenging in resource‐limited settings, and HBsAg assays remain rapid, affordable, and reliable, providing a consistent safety baseline. HBsAg testing may also help reduce hepatitis Delta infection, particularly in settings with high HBV prevalence.

The infectivity of ID‐NAT nonreactive HBsAg‐positive carriers is unclear. Low viremia reduces but does not eliminate transmission risk. Infections have occurred from HBsAg‐negative donors with undetectable viremia, even by sensitive ID‐NAT, suggesting an infectious dose as low as 3–32 HBV genome equivalents.^23^, ^24^, ^25^, ^27^ Direct assessment of infectivity in recipients is limited due to the systematic interception of HBsAg‐positive donations, and ethical and model constraints.^28^ Risk modeling estimated transfusion transmission at 5.5%–7.2% for red blood cells and 27%–31% for plasma, though uncertainty remains due to assumptions about the infectious dose (ID50: 316 virions).^2^ Furthermore, HBsAg‐positive donations missed by ID‐NAT screening are estimated to contribute a residual HBV transfusion‐transmission risk of 2%–6%.^29^ HBV DNA assays may not fully reflect viral genome integrity and/or presence of infectious virions. Defective particles from alternative splicing of HBV pgRNA have been found in patients, but this was not assessed here due to low viral loads.^30^ Nonenveloped capsids containing HBV nucleic acids have been described in vitro, though detection in vivo is challenging.^17^ For instance, strains B5129 and B5503 produced more naked capsids than complete virions in vitro, which may lower infectivity. This phenotype suggests impaired coupling between nucleocapsid maturation and envelopment. However, sequence analysis of both constructs, including comparison of pSKB5503 with the genotype‐matched pSKPO1125 clone displaying the opposite phenotype, revealed no notable differences in the PreS1 core‐binding region or the Core protein. Nevertheless, the involvement of uncharacterized mutations in PreS1 and/or Core cannot be excluded, as the molecular determinants governing capsid envelopment remain incompletely understood. Detection of HBV DNA after viral particle concentration, and recovery of complete genomes in 41% of donors, suggested the presence of potentially infectious virions in plasma. Among a small subset tested, two of five strains showed replicative ability comparable to HBsAg‐positive/HBV DNA‐positive controls when their genomes were transfected into cells. Beyond viral properties, host immune responses may also influence infectivity in HBsAg‐positive/ID‐NAT‐nonreactive donations, although the neutralizing activity of non‐anti‐HBs antibodies (e.g., anti‐PreS1) remains unclear.^31^, ^32^

Persistent and sustained HBsAg production, despite undetectable or minimal viral replication, may result from mutations affecting HBV replication or virion production, but not HBsAg expression. Examples of such mutations include changes in pgRNA transcription, nucleocapsid formation, or particle envelopment. Although no consistent pattern was found, the high mutation rates observed in the genomes of the studied strains supported this hypothesis. Functional analysis of selected mutations in the BCP revealed potentially disruptive changes, including an 8‐nucleotide deletion close to the pseudo‐TATA boxes in strain B6538, and a two‐nucleotide insertion in the pgRNA lnr element in strain B5594, which altered transcription efficiency. Meanwhile, dual mutations (G1862U/G1888U in strain SA2882 and C1860A/G1899A in strain B5594) likely destabilized the ε stem‐loop structure, which is essential for polymerase binding, pgRNA encapsidation, and reverse transcription initiation.

In the absence of dedicated functional assays, a defect in polymerase activity in case samples cannot be fully excluded, but appears unlikely given the complete conservation of residues essential for its function. Moreover, plasma RNA viremia was undetectable in a subset of case samples tested. Although plasma RNA viremia may be compatible with impaired HBV DNA synthesis, it does not independently demonstrate polymerase dysfunction.^33^ The absence of higher levels of plasma HBV RNA in the cases may suggest that there was no impaired conversion of HBV‐RNA to HBV‐DNA by the viral polymerase in the nascent virions, but rather reduced production of pre‐genomic HBV‐RNA by the infected hepatocytes. The absence of virion‐associated DNA may also result from impaired core maturation and defective particle assembly. Analysis of the HBV core protein revealed genotype‐specific variability in amino acids. Case strains of genotypes A1, A2, and E exhibited greater intra‐group diversity, indicating stronger selective pressures. In contrast, genotype D cases demonstrated greater conservation. Some case strains retained consensus‐like sequences, indicating that the HBsAg‐positive/HBV DNA‐negative phenotype is not strictly linked to increased core diversity. Amino acid substitutions occurred throughout the NTD and CTD domains, including residues that are critical for capsid assembly, virion production, and genome replication.^34^, ^35^, ^36^ Unique alterations in a domain critical for nucleic acid binding and genome encapsidation included cD153N/Y/Q in 4 genotype A strains and an RR insertion in one genotype C strain.^37^ Other alterations that are likely to impair the function of the core protein and viral replication include deletions at cE77 (in strains B7181 and B6197), cT147 (in strain PO2896), a 372‐nt deletion resulting in a 54‐aa C‐terminal extension (in strain B5598), and a premature termination at c184 (in strain SA5699). Additionally, multiple case‐specific and previously uncharacterized mutations, including cP45S, cP50H/A, cP79Q, cE113Q, cA131P and cP135L/Q/T, were absent in controls and could affect capsid stability or dynamics. Functional testing showed that most mutations were neutral. Only cN92T/cM93T and cS170F significantly reduced extracellular HBV DNA, albeit via mechanisms that remain unclear. Overall, mutations clustered near key functional regions, but evidence of replication defects was limited in vitro, likely requiring combinatorial or genotype‐specific effects.

Analysis of the complete S gene from 50 cases of HBV infection showed limited overall amino acid variability but genotype‐specific differences. This suggests possible variable selective pressures among viral lineages.^38^ Particular attention was given to the PreS1 domain, which is essential for virion formation and contains the core‐binding element (aa 103–124).^39^ Twelve of the case strains carried one to 3 substitutions in this region, which were mostly absent in the control group. These included Q104K, S109T, D114E, S115T, an R101 insertion, and changes near positions 118–120. These mapped to residues required for capsid docking and may modulate envelope‐capsid interactions, virion secretion efficiency, or viral fitness. The loss of the PreS1 and PreS2 start codons in strain B6078 would likely impair L and M protein synthesis, thereby disrupting the critical L:M:S ratio for virion assembly.

This study has several limitations. Only a subset of the full‐length genome and mutations in domains important for virion production were functionally tested. Further analyses are needed to determine how additional mutations influence virion assembly, including structural modeling of L‐HBs/core interactions and in‐depth phenotyping (e.g., capsid assembly and RNA‐binding assays). Furthermore, viral sequences obtained from plasma PCR may not accurately reflect intrahepatic viral gene expression. For example, the intracellular HBsAg retention observed in vitro for strain PO1125 did not correspond to the high HBsAg levels seen in vivo. Furthermore, circulating HBsAg largely originates from integrated HBV DNA rather than from cccDNA, which produces the encapsidated viral genomes analyzed.^40^ Finally, proper infectivity testing could not be conducted due to the lack of physiologically relevant models, as mentioned above.

In conclusion, HBsAg‐positive donors with undetectable or extremely low levels of HBV DNA exhibited chronic inactive HBV infection with low levels of circulating HBV DNA. No single molecular factor could explain the discrepancy between high HBsAg levels and minimal viremia. Instead, it is likely that diverse mutations in regulatory regions, the core region and possibly the PreS1 region likely contributed to this in a multifactorial manner. Further functional analyses are needed to clarify how combined mutations drive the HBsAg‐positive/HBV DNA‐negative phenotype. While certain strains exhibited diminished replication capacity, the infectivity of the majority remains uncertain, despite complete genomes being retrieved following particle concentration. These data support the continuation of HBsAg testing in blood screening programs where NAT sensitivity is suboptimal (e.g., pool testing) and anti‐HBc testing cannot be implemented.

## CONFLICT OF INTEREST STATEMENT

The authors have disclosed no conflicts of interest.

## Supporting information


**Figure S1.** HBV DNA testing of 50 blood donor samples with two ID‐NAT assays.
**Table S1.** HBV DNA reactivity of 75 HBsAg+ samples using four different assays.
**Table S2.** Amino acid mutations observed specifically in the core protein of the strains studied.
**Figure S2.** Alignment of HBV BCP/PC nucleotide sequences. Sequences from HBV‐infected blood donors with HBsAg levels >100 IU/mL and either ID‐NAT nonreactive or reactive results with HBV DNA <20 IU/mL were aligned against genotype‐matched consensus sequences obtained from HBsAg^+^/HBV DNA^+^ donor strains. HBV_D_ and HBV_E_ consensus sequences were identical. Nucleotides identical to the reference consensus are indicated by dots and deletions by dashes. ID‐NAT1 nonreactive sequences are shown in blue, and ID‐NAT1 reactive sequences are shown in black.
**Figure S3.** Alignment of Core amino acid sequences. Sequences from HBV‐infected blood donors with HBsAg levels >100 IU/mL and either ID‐NAT nonreactive or reactive results with HBV DNA <20 IU/mL were aligned against genotype‐matched consensus sequences obtained from HBsAg^+^/HBV DNA^+^ donor strains. Nucleotides identical to the reference consensus are indicated by dots and deletions by dashes. ID‐NAT1 nonreactive sequences are shown in blue, and ID‐NAT1 reactive sequences are shown in black.
**Figure S4.** Alignment of PreS1/PreS2 amino acid sequences. Sequences from HBV‐infected blood donors with HBsAg levels >100 IU/mL and either ID‐NAT nonreactive or reactive results with HBV DNA <20 IU/mL were aligned against genotype‐matched consensus sequences obtained from HBsAg^+^/HBV DNA^+^ donor strains. Amino acids identical to the reference consensus are indicated by dots and deletions by dashes. ID‐NAT1 nonreactive sequences are shown in blue, and ID‐NAT1 reactive sequences are shown in black. The putative core‐binding domain is highlighted in red.
**Figure S5.** Alignment of S amino acid sequences. Sequences from HBV‐infected blood donors with HBsAg levels >100 IU/mL and either ID‐NAT nonreactive or reactive results with HBV DNA <20 IU/mL were aligned against genotype‐matched consensus sequences obtained from HBsAg^+^/HBV DNA^+^ donor strains. Amino acids identical to the reference consensus are indicated by dots and deletions by dashes.
**Figure S6.** Alignment of Pol amino acid sequences. Sequences from HBV‐infected blood donors with HBsAg levels >100 IU/mL and either ID‐NAT nonreactive or reactive results with HBV DNA <20 IU/mL were aligned against genotype‐matched consensus sequences obtained from HBsAg+/HBV DNA+ donor strains. Amino acids identical to the reference consensus are indicated by dots and deletions by dashes. Functional domains within the Pol protein are represented by distinct colors. Critical motifs are highlighted in red, and essential functional residues are marked with an asterisk.
**Table S3.** Average Pol‐domain amino acid diversity in cases and controls stratified by HBV genotype.
**Figure S7.** HBV RNA semi‐quantification in plasmas of 37 case and 25 control samples. For clarity, individuals with negative RNA results (33 cases and 9 controls) are excluded from the figure. AU, arbitrary unit.

## Data Availability

The data that support the findings of this study are available from the corresponding author upon reasonable request.
