# Supplementary material for: Hepatitis B virus infection with high HBsAg and undetectable DNA levels in untreated blood donors: Viral mechanisms and transfusion safety implications
Source: Transfusion. 2026 Jul 20;66(10):1993–2006. doi: 10.1111/trf.70334 (PMC13674214; doi:10.1111/trf.70334)
Supplement: Supplementary file 1 [file TRF-66-1993-s001.docx]

**Revised Manuscript ID: 2805271**

**SUPPLEMENTARY MATERIALS**

Title: HBV infection with high HBsAg and undetectable DNA levels in untreated blood donors: viral mechanisms and transfusion safety implications

Authors: Maelenn Païline Delcourt, Marion Vermeulen, Aneta Kopacz, Manuela Miletic, Peter Gowland, Andrew Saville, Piotr Grabarczyk, Christoph Niederhauser, Jean-Michel Pawlotsky, Syria Laperche, Daniel Candotti

Table of contents

Supplementary Figure S1 …………………………………………………………………..2

Supplementary Table S1 ……………………………………………………………………3

Supplementary Table S2 ……………………………………………………………………4

Supplementary Figure S2 ………………………………………………………………..5-8

Supplementary Figure S3 ………………………………………………………………9-12

Supplementary Figure S4 …………………………………………………….…….…13-17

Supplementary Figure S5 ……………………………………………………………..18-25

Supplementary Figure S6 ……………………………………………………………..26-39

Supplementary Table S3 ……………………………………………….……………..40

Supplementary Figure S7 …………………………………………………………….41

**SUPPLEMENTARY TABLE S1.** HBV DNA reactivity of 75 HBsAg+ samples using four different assays.

| Procleix-Ultrio Plus/Elite  (ID-NAT1) | Cobas TaqScreen MPX Test V2.0  (ID-NAT2) | Cobas Taqman HBV | Nested-PCR  &  Sanger sequencing | Number  (%) |
| --- | --- | --- | --- | --- |
| Pos | Pos | Pos | Pos | 7 (9.3%) |
| Pos | Pos | Neg | Pos | 1 (1.3%) |
| Pos | Pos | Neg | Neg | 1 (1.3%) |
| Pos | Neg | Pos | Pos | 1 (1.3%) |
| Pos | Neg | Neg | Pos | 1 (1.3%) |
| Pos | NT | Pos | Pos | 23 (30.7%) |
| Pos | NT | Pos | Neg | 2 (2.7%) |
| Neg | Pos | Pos | Pos | 22 (29.4%) |
| Neg | Pos | Pos | Neg | 9 (12.0%) |
| Neg | Pos | Neg | Pos | 4 (5.3%) |
| **Neg** | **Neg** | **Pos** | **Neg** | **2 (2.7%)** |
| **Neg** | **Neg** | **Neg** | **Pos** | **2 (2.7%)** |

NT, not tested.

**SUPPLEMENTARY TABLE S2.** Amino acid mutations observed specifically in the core protein of strains studied.

| Core mutations reported associated with reduced viral particle production | | |  | Case-specific mutations introducing potential structural changes | |
| --- | --- | --- | --- | --- | --- |
| Sites | Reported functions  (references) | Case strains |  | Sites | Case strains |
| cP5L | Unclear | SA4546, **B5558** |  | 54-aa insertion | **B5558** |
| cD29H | Capsid assembly/virion production | **PO1125** |  | cT147Deletion | **PO2896** |
| cE77Deletion  N  A  Q | Unclear | **B7181**, B6197  **B5175**  **B5558**  **B5672**, **B6536** |  | cP50H | B5168, **B5129**, B5545 |
| cN92H  T | Capsid assembly/virion production | **SA018**, **SA5699**, **B5175**  **B5654** |  | cP79Q  cE113Q | **SA018**, B7112, B7169  **SA7504**, **B5175**, **B5672**, B6197, **B6536**, B7052, **B7181** |
| cM93T  cP130A  Q | Unclear  Capsid assembly/virion production | **B5654**  **B6536**  **B5129** |  | *cA131P* | **SA018**, SA5409, **SA5699**, **SA7504**, **B6197**, B7052 |
| I  cT147Deletion  A  cS155T | Unclear  HBV DNA replication | SA5256  **PO2896**  **B5129**, **B6197**  **PO2896** |  |  |  |
| cS170F | HBV DNA replication | **B6536**, B6156 |  |  |  |

```
		        10
Cons. A		GACTGGGAGGAGTTGGGGGAGGAGATTAGGTTAAAGGTCTTTGTATTAGGAGGCTGTAGGCATAAATTGGTCTGCGCACCATCATCATGCAACTTTTTCACCTC
SA018  A1	......................................G...........................................TCA...................
SA0127 A1	........................................................................................................
SA197  A1	........................................................................................................
SA1359 A1	......................................................................................C.................
SA2882 A1	......................................................................................C.................
SA4546 A1	.........................C........T.A...................................................................
SA4867	A1	......................................................................................C.................
SA5409 A1	..................................T.A.................................................T.................
SA5623 A1	........................................................................................................
SA5699 A1	..................................T.A...............................................T...................
SA6513 A1	........................................................................................................
SA6840	A1	........................................................................................................
SA7504 A1	......................................................................................C.................
SA8795 A1	....................T...C...........A.T.A.........................................TC....................
SA9624 A1	......................................................................................T.................
B6538  A1	..A...............................T--------.............................................................
PO2770 A2	............C.....................T.A.T..........................................G..C...................
SA8857 A2	........................................................................................................
SA9349 A2	............C....................................................................G..C...................
B5168  A2	............C............A.......................................................G..C...................
B5175  A2	............C....................................................................G..C...................
B5558  A2	............C....................................................................G..C...................
B5654  A2	............C............C........T.A.T..........................................G..C...................
B5672  A2	............C....................................................................G..C...................
B6536  A2	............C.......................A.T..........................................G..C...................
B7227  A2	............C....................................................................G..C...................
B7086  A3	.........................C........T.A..............................................T....................


Cons. A		TGCCTAAT-CATCTCTTGTA--CATGTCCCACTGTTCAAGCCTCCAAGCTGTGCCTTGGGTGGCTTTGGGGCATGGACATTGACCCTTATAAAGAATT
SA018  A1	........-...........--............................................................................
SA0127 A1	........-...........--.....................................A......................................
SA197  A1	........-...........--.....................................A......................................
SA1359 A1	........-...........--................................................A...........................
SA2882 A1	........-...........--...........T.........................T......................................
SA4546 A1	........-...........--...............................................................T............
SA4867 A1	........-...........--...........T.........................A......................................
SA5409 A1	........-...........--.....................................A......................................
SA5623 A1	........-...........--.....................................A......................................
SA5699 A1	........-...........--.....................................T......................................
SA6513 A1	........-...........--.....................................A......................................
SA6840 A1	........-...........--............................................................................
SA7504 A1	........-...........--.....................................A......................................
SA8795 A1	........-...........--...........C................................................................
SA9624 A1	........-...........--..........................T.........A.......................................
B6538  A1	........-...........--...........T.........................A......................................
PO2770 A2	........-...........--.......T.....................................A..............................
SA8857 A2	........-...........--....................................A.......................................
SA9349 A2	........-...........--............................................................................
B5168  A2	........T...........--............................................................................
B5175  A2	........-...........--......T.......................................A.............................
B5558  A2	........-...........--.......T.....................................A.................T............
B5654  A2	........-...........--............................................................................
B5672  A2	........-..........GTT............................................................................
B6536  A2	.......AT..........T--.......T.....................................A..A...........................
B7227  A2	........-..........T--.......T.....................................A..............................
B6027  A3	........-...........--...........C................................................................
B7086  A3	........-...........--...........T................................................................
```

```
Cons. B/C	GAGTGGGAGGAGTTGGGGGAGGAGATTAGGTTAAAGGTCTTTGTACTAGGAGGCTGTAGGCATAAATTGGTCTGTTCACCAGCACCATGCAACTTTTTCACCTC
B5129 C		..C..........................A.........................................................G..G.............
B5168 B		.......................................................................G..C......T......................
B7081 B		..................................T.A........T..........................................................

Cons. B/C	TGCCTAATCATCTCATGTTCATGTCCTACTGTTCAAGCCTCCAAGCTGTGCCTTGGGTGGCTTTGGGGCATGGACATTGACCCGTATAAAGAATT
B5129 C		................................................................A..................T...........
B5168 B		...............................................................................................
B7081 B		...............................................................................................


Cons.   D/E	GACTGGGAGGAGTTGGGGGAGGAGATTAGATTAAAGGTCTTTGTACTAGGAGGCTGTAGGCATAAATTGGTCTGCGCACCAGCACCATGCAAC--TTTTTCACC
B5594    D	.........................C...G....T.A........................................................AC.........
PO2896   D	.............................G........................................................C......--.........
SA5256   D	.............................................................................................--.........
B5503    D	.............................................T....................................T..........--.........
B5545    D	..................................T.A........................................................--.........
B6052    D	.............................................T...............................................--.........
B7169    D	........................C....................................................................--.........
B7181    D	.............................G...............................................................--.........
CR254029 D	.............................................................................................--.........
CR417082 D	.............C....................T.A........................................................--.........
PO1125   D	............C................G....T.A.T......T...............................................--.........
B6156    E	.............................G...............................................................--.........
B6197    E	.............................G....................................................T..........--.........
B6682    E	.............................................................................................--.........
B7047    E	.............C...............................................................................--.........
B7052    E	.............................G..............................................T................--.........
B7112    E	........................C....................................................................--.........
B7152    E	.............................G...............................................................--.........


Cons.   D/E	TCTGCCTAATCATCTCTTGTTCATGTCCTACTGTTCAAGCCTCCAAGCTGTGCCTTGGGTGGCTTTAGGGCATGGACATTGACCCTTATAAAGAATT
B5594    D	..............................A......................................A...........................
PO2896   D	................A..............................................................C.................
SA5256   D	..................................................................G..................G...........
B5503    D	............C....................................................................................
B5545    D	..................................................................G..................G...........
B6052    D	.................................................................................................
B7169    D	................A..........................T............A......................C.................
B7181    D	.................................................................................................
CR254029 D	.....................................................................A...........................
CR417082 D	..............................T......................................................G...........
PO1125   D	....................A............................................................................
B6156    E	.................................................................................................
B6197    E	.....................................................................................G...........
B6682    E	.................................................................................................
B7047    E	.....................................................................A...........................
B7052    E	.....................................................................A...........................
B7112    E	.................................................................................................
B7152    E	..................................................................G..............................
```

**SUPPLEMENTARY FIGURE S2. Alignment of HBV BCP/PC nucleotide sequences.** Sequences from HBV-infected blood donors with HBsAg levels >100 IU/mL and either ID-NAT nonreactive or reactive results with HBV DNA <20 IU/mL were aligned against genotype-matched consensus sequences obtained from HBsAg⁺/HBV DNA⁺ donor strains. HBV_D_ and HBV_E_ consensus sequences were identical. Nucleotides identical to the reference consensus are indicated by dots and deletions by dashes. ID-NAT1 nonreactive sequences are shown in blue, and ID-NAT1 reactive sequences are shown in black.

```
		        10        20        30        40        50        60        70        80        90       100
Cons. A1	MDIDPYKEFGATVELLSFLPSDFFPSVRDLLDTASALYREALESPEHCSPHHTALRQAILCWGELMTLATWVGNNLEDPASRDLVVNYVNTNMGLKIRQL
SA018		.....................................F.D..................V...................Q............H........
SA0127	....................................................................................................
SA197		....................................................................................................
SA2882	...........S......................A...................I.............................................
SA4546	....L..........................................................................T...............R....
SA5409	...............................................................DVTN......T..D......R................
SA5623	....................................................................................................
SA5699	...............................................G......I.......V............................H........
SA6513	....................................................................................................
SA7504	.................................................................T..G...............................


		       110       120       130       140       150       160       170       180
Cons. A1	LWFHISCLTFGRETVLEYLVSFGVWIRTPPAYRPPNAPILSTLPETTVVRRRDRGRSPRRRTPSPRRRRSQSPRRRRSQSRESQC
SA018		..............................P......................................................
SA0127	.....................................................................................
SA197		.....................................................................................
SA2882	......SI............................................N.............................P..
SA4546	.....................................................................................
SA5409	..............................P......................................................
SA5623	.....................................................................................
SA5699	..............................P.....................Y..............................*.
SA6513	.....................................................................................
SA7504	............Q.................P...................Q..................................
```

```
		        10        20        30        40        50        60        70        80        90       100
Cons. A2	MDIDPYKEFGATVELLSFLPSDFFPSVRDLLDTASALYREALESPEHCSPHHTALRQAILCWGELMTLATWVGNNLEDPASRDLVVNYVNTNMGLKIRQL
PO2770	...........S.........................C.D....S..................DVTN......T..Q.......................
SA8857	....................................................................................................
SA9349	....................................................................................................
B5168		..................................T..F...........H...............TN.G...............................
B5175		.......................Y..............................I...V.................N..............H........
B5654		...........S...........................D.......................D.TN......S.................TT.......
B5672		....................N..................D..................V.......A..N......Q.......................
B6536		...........S...I....L.....A...............................A...V..T.......G..Q.............N.........


		       110       120       130       140       150       160       170       180
Cons. A2	LWFHISCLTFGRETVLEYLVSFGVWIRTPPAYRPPNAPILSTLPETTVVRRRDRGRSPRRRTPSPRRRRSQSPRRRRSQSRESQC
PO2770	....L..I..........................................................................P..
SA8857	.....................................................................................
SA9349	.....................................................................................
B5168		.............I.......................................................................
B5175		............Q........................................................................
B5654		M..........K......................Q...............Q.Q.H..............................
B5672		............QI....................................S.N................................
B6536		............Q..V.............A...........L......I................C...F...............


		        10        20        30        40        50        60        70        80        90       100
Cons. C	MDIDPYKEFGASVELLSFLPSDFFPSIRDLLDTASALYREALESPEHCSPHHTALRQAILCWGELMNLATWVGSNLEDPASRELVVSYVNVNMGLKIRQL
B5129		.............N...................................H..........................D.............I.........


		       110       120       130       140       150       160       170       180
Cons. C	LWFHISCLTFGRETVLEYLVSFGVWIRTPPAYRPPNAPILSTLPETTVVRRRG--RSPRRRTPSPRRRRSQSPRRRRSQSRESQC
B5129		.............................Q................A...P.NRR....................G.........


		        10        20        30        40        50        60        70        80        90       100
Cons. D	MDIDPYKEFGASVELLSFLPSDFFPSVRDLLDTASALYREALESPEHCSPHHTALRQAILCWGELMTLATWVGVNLEDPASRDLVVSYVNTNMGLKFRQL
B5594		....................T..................D.......................D....................................
B6612		.......................................D........T.........................................N.........
PO2896	................................................T...................................................
SA5256	.......................................Q............................................................
B5503		....................T..................D.......................D....................................
B5545		...........T........F........F...................H..................................................
B6052		.........................................................................G.....T....................
B7169		...........T..................................................................Q.....................
B7181		....................V.....................................T..............A..-.......................
PO1125	............................H....................A..................................................


		       110       120       130       140       150        160       170       180
Cons. D	LWFHISCLTFGRETVIEYLVSFGVWIRTPPAYRPPNAPILSTLPETTVVRRRGRSPRRRRTPSPRRRRSQSPRRRRSQSRESQC
B5594		..................................T.................C................K..............
B6612		...............T..........................I...C........L............................
PO2896	...............L..............................-...Q...T.............................
SA5256	.............................I......................................................
B5503		....V.............................T...............................................K.
B5545		....................................................................................
B6052		........M......L................................I...............................G...
B7169		..Y.L..........L.............................................................K......
B7181		............Q................................C..I...................................
PO1125	............D.......................................................................


		        10        20        30        40        50        60        70        80        90       100
Cons. E	MDIDPYKEFGATVELLSFLPSDFFPSVRDLLDTASALYRDALESPEHCSPHHTALRQAILCWGELMTLATWVGVNLEDPASRDLVVSYVNTNMGLKFRQL
B6156		..................................................................N......A.....S....................
B6197		...........S........Q..................E.........A.......................A..-.............E.........
B7052		....................A....................................................A................N.........
B7102		....................................................................................................
B7112		....................A......K.........F.........................D..N............Q..E.................


		       110       120       130       140       150       160       170       180
Cons. E	LWFHISCLTFGRETVIEYLVSFGVWIRTPPAYRPPNAPILSTLPENTVVRRRGRSPRRRTPSPRRRRSQSPRRRRSQSPASQC
B6156		....V..............................................................F...............
B6197		....T......KQ..V..............P...L......I...TA....................................
B7052		............Q..T..............P....................................................
B7102		...................................................................................
B7112		............DI.L.............................HS....................................
```

**SUPPLEMENTARY FIGURE S3. Alignment of Core amino acid sequences.** Sequences from HBV-infected blood donors with HBsAg levels >100 IU/mL and either ID-NAT nonreactive or reactive results with HBV DNA <20 IU/mL were aligned against genotype-matched consensus sequences obtained from HBsAg⁺/HBV DNA⁺ donor strains. Nucleotides identical to the reference consensus are indicated by dots and deletions by dashes. ID-NAT1 nonreactive sequences are shown in blue, and ID-NAT1 reactive sequences are shown in black.

```
		        10        20        30        40        50        60        70        80        90       100
Cons. A1/A3	MGGWSSKPRKGMGTNLSVPNPLGFFPDHQLDPAFGANSNNPDWDFNPIKDHWPQANQVGVGAFGPGFTPPHGGVLQWSPQAQGILATVPAVPPPASTNRQ
SA018		........................L..............................................................L.T..........
SA0127	.....A...................................................................I.........T................
SA197		.....A...................................................................I.........T................
SA0763	........................L..............................................................L.T..........
SA2882	........................L......................V....................................................
SA4546	.....A...................................................................................S...T......
SA5623	.....A...................................................................I.........T................
SA5699	.....A.......................................................................................T......
SA5753	........................L.........................Q....................................L.T..........
SA6513	.....A...................................................................I.........T................
SA7504	........................................Q..............................................A.V..........
SA8795	.....A...................................................................................P..........
B5505		...............................................V....................................................
B5631		...RL...........................................................................................A...
B6027		....LP.................................K.............E...............................T..............
B6538		....................................................................................................
B7086		....LP.................................K.............E...............................T....S.........
```

```
		       110       120       130       140       150       160       170       180
Cons. A1/A3	SGRQPTPISPPLRDSHPQAMQWNSTAFHQALQDPRVRGLYFPAGGSSSGTLNPVPNIASHISSISSRTGDPAPN
SA018		............................R.....................................T....V..
SA0127	........................................................................L.
SA197		........................................................................L.
SA0763	............................T.....................................T....V..
SA2882	..................................K.....P..........................I......
SA4546	...................R....................L...............T...............L.
SA5623	........................................................................L.
SA5699	........................K...............................T...............L.
SA5753	..................................................................K....V..
SA6513	........................................................................L.
SA7504	........................................L.........H...L......L....K.....L.
SA8795	........................................................................L.
B5505		.........................T................................................
B5631		........................................................................S.
B6027		..................................................V..A..T................I
B6538		................................................E.........................
B7086		..................................................V..A...................I

		        10        20        30        40        50        60        70        80        90       100
Cons. A2	MGGWSSKPRKGMGTNLSVPNPLGFFPDHQLDPAFGANSNNPDWDFNPIKDHWPAANQVGVGAFGPGLTPPHGGILGWSPQAQGILTTVSTIPPPASTNRQ
SA8857	....................................................................................................
SA9349	....................................................................................................
B5168		......T...........................................................F.....S.................V.........
B5175		.............R........................................S...........F......V..........................
B5558		........................................Q...L.....................F......V..........................
B5654		......T.............................................................................................
B5672		..................................................................F......V..........................
B6536		....................................................................................................
B7227		....................................................................................................
```

```
		       110       120       130       140       150       160       170       180
Cons. A2	SGRQPTPISPPLRDSHPQAMQWNSTAFHQALQDPRVRGLYFPAGGSSSGTVNPAPNIASHISSISARTGDPVTN
SA8857	..........................................................................
SA9349	..........................................................................
B5168		...K.....................P..................S.........R...................
B5175		..............................V...........................................
B5558		..............T...........................................................
B5654		.............E....................K.......................................
B5672		..........................................................................
B6536		..........................................................................
B7227		........T........H.T............N.........................................


		        10        20        30        40        50        60        70        80        90       100
Cons. B	MGGWSSKPRKGMGTNLSVPNPLGFFPDHQLDPAFKANSENPDWDLNPHKDNWPDANKVGVGAFGPGFTPPHGGLLGWSPQAQGILTTVPAAPPPASTNRQ
B7081		...............................................Y....................................................


		       110       120       130       140       150       160       170       180
Cons. B	SGRQPTPLSPPLRDTHPQAMQWNSTTFHQTLQDPRVRALYFPAGGSSSGTVSPAQNTVSAISSILSKTGDPVPN
B7081		.........................................................A.....TF.........
```

```
		        10        20        30        40        50        60        70        80        90       100
Cons. C	MGGWSSKPRQGMGTNLSVPNPLGFFPGHQLDPAFGANSNNPDWDFNPNKDQWPAANQVGVGSFGPGFTPPHGSLLGWSPQAQGILTTVPAAPPPASTNRQ
B5129		.........K................D.................L........E.T...A.A..........G................TS.........


		       110       120       130       140       150       160       170       180
Cons.C	SGRQPTPISPPLRDSHPQAMQWNSSTFHQALLDPRVRGLYFPAGGSSSGTVNPVPTTASPISSIFSRTGDPAPN
B5129		........................T..............................I................Q.
```

```
		        10        20        30        40        50        60        70        80        90       100
Cons. D	MGQNLSTSNPLGFFPDHQLDPAFRANTANPDWDFNPNKDTWPDANKVGAGAFGLGFTPPHGGLLGWSPQAQGILQTLPANPPPASTNRQSGR-QPTPLSP
B5594		...D......................................N..............................I..M............T..-.......
PO1692	.........................................................................................T..-.......
PO2896	.........................................................................I..................-.......
SA5256	..................................................................N.....................R...-.......
B5503		...........................T........K..P.................................I...............PR.-.......
B5545		......................................................................................I.....-.......
B6052		........................................................................T.....T.............-.......
B6078		T...........................................................................................-.......
B7169		...........................T.............................................I..................-.......
B7181		...........................N.............................................I..................-.......
B7197		............................................S.................................T.............-.......
CR417082	..............................................................................T.............-.......
PO1125	.........................................................................I................S.R.......
```

```
		       110       120       130       140       150       160
Cons.D	PLRNTHPQAMQWNSTTFHQTLQDPRVRGLYFPAGGSSSGTVNPVPTTASPISSIFSRIGDPALN
B5594		...D...................................P........................
PO1692	.......................................A..........L.............
PO2896	................................................................
SA5256	..................................................L.............
B5503		...D............................................................
B5545		.......................................A..........L.............
B6052		...............................................V.H..............
B6078		.........T..................................L.....L.............
B7169		................................................................
B7181		................................................................
B7197		...............................................V.H..............
CR417082	......................................E..T........L...........Q.
PO1125	...D..............K.............................................
```

```
		        10        20        30        40        50        60        70        80        90       100
Cons. E	MGLSWTVPLEWGKNMSTTNPLGFFPDHQLDPAFRANTRNPDWDHNPNKDHWTEANKVGVGAFGPGFTPPHGGLLGWSPQAQGMLKTLPADPPPASTNRQS
B6156		.................................K...S.....Q.......I.G.............................Q................
B6197		..............L...................................................................IQ.....N..........
B6507		...........................................Q.......K.............L................T...............R.
B6662		........................................................................................T...........
B7047		.................................................................V..................................
B7052		.................................K............K................S..................T.................
B7102		.................................K..................................................................
B7112		..................................................................................................R.
B7152		          .......................................R..QN..............................................
```

```
		       110       120       130       140       150       160       170
Cons. E	GRQPTPITPPLRDTHPQAMQWNSTTFHQALQDPRVRGLYFPAGGSSSGTVNPVPTTASLISSIFSRIGDPAPN
B6156		..................V.....K......N.......L.................................
B6197		..................IR...........N.......L......................T..........
B6507		.......................................L.............................L...
B6662		............................................F....R.......................
B7047		........................K..........K.................................L...
B7052		.................................K.......................................
B7102		.........................................................................
B7112		.................................K...................................L...
B7152		.................V.R...........N-----HPHT.....................L..........
```

**SUPPLEMENTARY FIGURE S4. Alignment of PreS1/PreS2 amino acid sequences.** Sequences from HBV-infected blood donors with HBsAg levels >100 IU/mL and either ID-NAT nonreactive or reactive results with HBV DNA <20 IU/mL were aligned against genotype-matched consensus sequences obtained from HBsAg⁺/HBV DNA⁺ donor strains. Amino acids identical to the reference consensus are indicated by dots and deletions by dashes. ID-NAT1 nonreactive sequences are shown in blue, and ID-NAT1 reactive sequences are shown in black. The putative core-binding domain is highlighted in red.

```
		        10        20        30        40        50        60        70        80        90       100
Cons.A1/A3	MENITSGFLGPLLVLQAGFFLLTRILTIPQSLDSWWTSLNFLGGSPVCLGQNSQSPTSNHSPTSCPPICPGYRWMCLRRFIIFLFILLLCLIFLLVLLDY
SA018		.......................K............................................................................
SA0127	....................................................................................................
SA197		....................................................................................................
SA0763	....................................................................................................
SA2882	....................................................................................................
SA4546	...................S................................................................................
SA5623	....................................................................................................
SA5699	............................................P...R...................................................
SA5753	.......................K............................................................................
SA6513	....................................................................................................
SA7504	....................................................................................................
SA8795	.........................................P.........................T................................
B5505		....................................................................................................
B5631		............................................P.......................................................
B6027		....................................................................................................
B6538		....................................................................................................
B7086		............................................P.......................................................


		       110       120       130       140       150       160       170       180       190       200
Cons. A1/A3	QGMLPVCPLIPGSTTTSTGPCKTCTTPAQGNSMFPSCCCTKPTDGNCTCIPIPSSWAFAKYLWEWASVRFSWLSLLVPFVQWFVGLSPTVWLSVIWMMWY
SA018		.............................................................................................A......
SA0127	....................................................................................................
SA197		....................................................................................................
SA0763	....................................................................................................
SA2882	....................................................................................................
SA4546	....................................................................................................
SA5623	....................................................................................................
SA5699	............................................................F.......................................
SA5753	....................................................................................................
SA6513	....................................................................................................
SA7504	....................................................................................................
SA8795	....................................................................................................
B5505		................................T..........A................F................................A......
B5631		.....................R..............................................................................
B6027		.....................R....................................G........................A................
B6538		.............................................................................................A......
B7086		.....................R....................................G........................A................
```

```
		       210       220
Cons. A1/A3	WGPSLYNILSPFIPLLPIFFCLWYI
SA018		.........................
SA0127	......D..................
SA197		.........................
SA0763	.........................
SA2882	.........................
SA4546	..R......................
SA5623	.........................
SA5699	.........................
SA5753	.........................
SA6513	.........................
SA7504	.........................
SA8795	.........................
B5505		..........R..............
B5631		.......................F.
B6027		.........R...............
B6538		.........................
B7086		...N.....................
```

```
		        10        20        30        40        50        60        70        80        90       100
Cons. A2	MENITSGFLGPLLVLQAGFFLLTRILTIPQSLDSWWTSLNFLGGSPVCLGQNSQSPTSNHSPTSCPPICPGYRWMCLRRFIIFLFILLLCLIFLLVLLDY
SA8857	....................................................................................................
SA9349	....................................................................................................
B5168		...........................................E........................................................
B5175		....................................................................................................
B5558		....................................................................................................
B5654		..............................N........S..............C.............................................
B5672		.................................L........EE....R.....C............T.....L..........................
B6536		.................................L.................................T................................
B7227		.......................................S....P.......................................................


		       110       120       130       140       150       160       170       180       190       200
Cons. A2	QGMLPVCPLIPGSTTTSTGPCKTCTTPAQGNSMFPSCCCTKPTDGNCTCIPIPSSWAFAKYLWEWASVRFSWLSLLVPFVQWFVGLSPTVWLSAIWMMWY
SA8857	....................................................................................................
SA9349	....................................................................................................
B5168		....................................................................................................
B5175		.....................................................................................P..............
B5558		....................................................................................................
B5654		.....T.......................................................................................V......
B5672		.............................................................................................V......
B6536		.............................................................................................V......
B7227		............................................................................................LV......


		       210       220
Cons. A2	WGPSLYSIVRPFIPLLPIFFCLWVYI
SA8857	.........S................
SA9349	.........S................
B5168		.........S................
B5175		..........................
B5558		...R.....S................
B5654		..........................
B5672		.A......LS.............A..
B6536		...N..N..S................
B7227		.A.N.....S................


		        10        20        30        40        50        60        70        80        90       100
Cons. B	MENIASGLLGPLLVLQAGFFLLTKILTIPQSLDSWWTSLNFLGGTPVCLGQNSQSQISSHSPTCCPPICPGYRWMCLRRFIIFLCILLLCLIFLLVLLDY
B7081		....................................................................................................


		       110       120       130       140       150       160       170       180       190       200
Cons. B	QGMLPVCPLIPGSSTTSTGPCKTCTTPAQGTSMFPSCCCTKPTDGNCTCIPIPSSWAFAKYLWEWASVRFSWLSLLVPFVQWFVGLSPTVWLSVIWMMWF
B7081		...................S.R...........................................................................I.Y


		       210       220
Cons. B	WGPSLYNILSPFMPLLPIFFCLWVYI
B7081		..........................


		        10        20        30        40        50        60        70        80        90       100
Cons. C	MESTTSGFLGPLLVLQAGFFLLTRILTIPQSLDSWWTSLNFLGGAPTCPGQNLQSPTSNHSPTSCPPICPGYRWMCLRRFIIFLFILLLCLIFLLVLLDY
B5129		..N..............V......V..................E..R.....S...............................................


		       110       120       130       140       150       160       170       180       190       200
Cons.C	QGMLPVCPLLPGTSTTSTGPCKTCTIPAQGTSMFPSCCCTKPSDGNCTCIPIPSSWAFARFLWEWASVRFSWLSLLVPFVQWFVGLSPTVWLSVIWMMWY
B5129		..........................................................V.................A.......................


		       210       220
Cons.C	WGPSLYNILNPFLPLLPIFFCLWVYI
B5129		.........S..I.............
```

```
		        10        20        30        40        50        60        70        80        90       100
Cons. D	MENITSGFLGPLLVLQAGFFLLTRILTIPQSLDSWWTSLNFLGGTTVCLGQNSQSPTSNHSPTSCPPTCPGYRWMCLRRFIIFLFILLLCLIFLLVLLDY
B5594		.......S............................................................................................
PO1692	....................................................................................................
PO2896	.............................................................................................G......
SA5256	...........................................E........................................................
B5503		...........................................................................Y........................
B5545		.......L................................................I..........................................F
B6052		....................................................................................................
B6078		....................................................................................................
B7169		.......................................S...........................I................................
B7181		........................................S...........................................................
B7197		...................S................................................................................
CR417082	.......................................S.....................................................G......
PO1125	.......................K............................................................................


		       110       120       130       140       150       160       170       180       190       200
Cons.D	QGMLPVCPLIPGSSTTSTGPCRTCTTPAQGTSMYPSCCCTKPSDGNCTCIPIPSSWAFGKFLWEWASARFSWLSLLVPFVQWFVGLSPTVWLSVIWMMWY
B5594		.................A........T......F.......................................N...Q..........I...L.......
PO1692	........................M.T.........................................................................
PO2896	.................V........TV........................................................................
SA5256	.........................................................................N..........................
B5503		...................................................................................A................
B5545		........................M.A..R......................................................................
B6052		....................................................................................................
B6078		........................M.T.........................................................................
B7169		.................A........T.........................................................................
B7181		................................................................................................T...
B7197		..................................................................................C.........L.......
CR417082	....................................................................................................
PO1125	.................V........TV.......................................................A....I...........
```

```
		       210       220       230
Cons.D	WGPSLYSILSPFLPLLPIFFCLWVYI
B5594		...N......................
PO1692	..........................
PO2896	..........................
SA5256	..........................
B5503		......R...................
B5545		.......................A..
B6052		..........................
B6078		..........................
B7169		...R.C....................
B7181		......N...................
B7197		...KVF....................
CR417062	.....
PO1125	...N..NT..................


		        10        20        30        40        50        60        70        80        90       100
Cons. E	MESITSGFLGPLLVLQAGFFLLTKILTIPQSLDSWWTSLNFLGGAPVCLGQNSQSPTSNHSPTSCPPICPGYRWMCLRRFIIFLFILLLCLIFLLVLLDY
B6156		..G.....................................................I...........................................
B6197		.......................................S......A.....................................................
B6507		....................S...............................................................................
B6662		....................................................................................................
B7047		..T.................................................................................................
B7052		........................................................I...........................................
B7102		........................................................I...........................................
B7112		........................................................I...............H...........................
B7152		..........................................................S.........................................


		       110       120       130       140       150       160       170       180       190       200
Cons. E	QGMLPVCPLIPGSSTTSTGPCRTCTTLAQGTSMFPSCCCSKPSDGNCTCIPIPSSWAFGKFLWEWASARFSWLSLLVPFVQWFAGLSPTVWLSVIWMMWY
B6156		........................................................................................I...........
B6197		....................................................................................................
B6507		.................................I.............................G....................................
B6662		....................................................................................................
B7047		..........................I.........................................................................
B7052		....................................................................................................
B7102		...........................................E........................................................
B7112		H..............................................................G............A.......................
B7152		....................................................................................................

		       210       220
Cons. E	WGPSLYNILSPFIPLLPIFFCLWVYI
B6156		..........................
B6197		..........................
B6507		.......................A..
B6662		..........................
B7047		..........................
B7052		..........................
B7102		..........................
B7112		.............L............
B7152		..........................
```

**SUPPLEMENTARY FIGURE S5. Alignment of S amino acid sequences.** Sequences from HBV-infected blood donors with HBsAg levels >100 IU/mL and either ID-NAT nonreactive or reactive results with HBV DNA <20 IU/mL were aligned against genotype-matched consensus sequences obtained from HBsAg⁺/HBV DNA⁺ donor strains. Amino acids identical to the reference consensus are indicated by dots and deletions by dashes.

```
									Terminal protein domain
	__________________________________________________________________________________________________________________________________
	        10        20        30        40        50        60    *   70        80        90       100       110       120       130
Cons. A1	MPLSYQHFRKLLLLDDETEAGPLEEELPRLADEDLNRRVAEDLNLGNLNVSIPWTHKVGNFTGLYSSTVPIFNPEWQTPSFPKIHLHEDIINRCQQFVGPLTVNEKRRLKLIMPARFYPNSTKYLPLDKG
SA018	......................................................................N...D.......................................................
SA0127	..................................................................................................................................
SA197	....C.............................................................................................................................
SA2882	...................V......................................................................S............................I..........
SA4546	......................................................................V...............Q...........................................
SA5623	..................................................................................................................................
SA5699	................D.....................................................V...........................................................
SA6513	..................................................................................................................................
SA7504	...............N..................................................................................................................
SA0763
SA5753
SA8795
B5505
B5631
B6538


	                                                                                               Spacer domain
	__________________________________________________________________________________________________________________________________
	       140       150       160       170       180       190       200       210       220       230       240       250       260
Cons. A1	IKPYYPDQVVNHYFQTRHYLHTLWKAGILYKRETTRSASFCGSPYSWEQELHHGRLVIKTSQRHGDESFCSQPSGILSRSSVGPCIRSQFKQSRLGLQPHQGPLATSQPGRSGSIRARVHSPTRRCFGVE
SA018	.................................S.....................................K....P....T..........................S.....................
SA0127	.........................................................S..........................................................P........Y....
SA197	.........................................................S..........................................................P........Y....
SA2882	.......H...........................................................P........P......................R......G.......................
SA4546	.........................................................SQA............................K...........................P.............
SA5623	.........................................................S..........................................................P........Y....
SA5699	.........................................................SQ.........................................................P.............
SA6513	.........................................................S..........................................................P........Y....
SA7504	..................................S......................................................................S........F.P.............
SA0763	                                                                  .....K....P...............................S...........P.........
SA5753	                                                                  .....K....P....T..............F......M....S.....................
SA8795	                                                                  ................................................................
B5505	                                                                  .P................................R.............................
B5631	                                                                  ....P...................................................S.......
B6538	                                                                  .P..............................................................


	__________________________________________________________________________________________________________________________________
	       270       280       290       300       310       320       330       340       350       360       370       380       390
Cons. A1	PSGSGHIGHSASSSSSCLHQSAVRKAAYSHLSTSKRQSSSGHAVEFHSFPPSSARSQSQGPVFSCWWLQFRNTQPCSKYCLSHLVNLLEDWGPCVEHGEHHIRIPRTPARVTGGVFLVDKNPHNTAESRL
SA018	.........I.N......................................Q.....................................D.....A...................................
SA0127	........Y......................................R.........................E........................................................
SA197	........Y......................................R.........................E........................................................
SA2882	..............................................................S...............................A...................................
SA4546	........Y..I...H..................E......Q....................S...................................................................
SA5623	........Y......................................R.........................E........................................................
SA5699	........Y......H..........T...................Q......................................D............................................
SA6513	........Y......................................R.........................E........................................................
SA7504	........Y......................................................A..................................................................
SA0763	.........I.N......................................H.....................................D.....A...................................
SA5753	........NI.N...............H..................................................................A...................................
SA8795	........Y..T....................................................................I.............A...................................
B5505	..........T....................................N..............................................A...................................
B5631	........Y....A....R..............................S......P...........................................Y.............................
B6538	.............T................................................................................A...................................


	                                                         Reverse Transcriptase (RT) Domain
	__________________________________________________________________________________________________________________________________
	       400       410       420       430*      440       450       460       470       480       490       500       510       520
Cons. A1	VVDFSQFSRGLTRVSWPKFAVPNLQSLTNLLSSNLSWLSLDVSAAFYHIPLHPAAMPHLLIGSSGLPRYVARLSSNSRIHNNQYGTLQNLHDSCSRQLYVSLMLLYKTYGWKLHLYSHPIILGFRKIPMG
SA018	..........I...................................................T...S............N..................................................
SA0127	.................................................................................Y................................................
SA197	.................................................................................Y................................................
SA2882	..........V.......................................................S...............................................................
SA4546	..................................................................................................................................
SA5623	.................................................................................Y................................................
SA5699	..........P.......................................................................................................................
SA6513	.................................................................................Y................................................
SA7504	..................................................................................E...............................................
SA0763	..........I...................................................T...SG...........N..................................................
SA5753	..........V...................................................T...S............N..................................................
SA8795	..........I........................................................G...........N..............................R...................
B5505	..........I.......................................................S.......P.......................................................
B5631	..........P....................................................................N...H....D.........................................
B6538	..........I.......................................................S...............................................................


	                                                         Reverse Transcriptase (RT) Domain
	__________________________________________________________________________________________________________________________________
	       530       540       550  **   560       570       580       590       600       610       620       630       640       650
Cons. A1	VGLSPFLLAQFTSAICSVVRRAFPHCLAFSYMDDVVLGAKSVQHLESLYTAVTNFLLSLGIHLNPNKTKRWGYSLNFMGYVIGSWGTLPQDHIVQKIKHCFRKLPVNRPIDWKVCQRIVGLLGFAAPFTQ
SA018	...................................................................................G.........I....C...............................
SA0127	..........................................R.....................................I.....S......RR...................................
SA197	......................................................................................S......RR...................................
SA2882	.............................................................................................I....................................
SA4546	.............................................................................................I....................................
SA5623	......................................................................................S......RR...................................
SA5699	.............................................................................................RH...................................
SA6513	......................................................................................S......RR...................................
SA7504	.......................................................................................................I..........................
SA0763	.........................................................................................A..I....C............
SA5753	...............................................................H.....................S...........C......F.....
SA8795	................................................................................................RC............
B5505	............................................................................................I.................
B5631	................................................................H.............................................
B6538	............................................................................................I.................


	                                                                          RNase H Domain
	__________________________________________________________________________________________________________________________________
	       660       670       680       690       700 *     710       720       730*      740         *       760       770       780
Cons. A1	CGYPALMPLYACIQAKQAFTFSPTYKAFLSKQYMNLYPVARQRPGLCQVFADATPTGWGLAIGHQRMRGTFVAPLPIHTAELLAACFARSRSGAKLIGTDNSVVLSRKYTSFPWLLGCTANWILRGTSFV
SA018	.....................................................A............................................................................
SA0127	..............................Q..............................V....................................................................
SA197	..............................Q..............................V....................................................................
SA2882	..................................................................................................................................
SA4546	.............................NQ............S......................................................................................
SA5623	..............................Q..............................V....................................................................
SA5699	.............................NQ............S..................................................Q...................................
SA6513	..............................Q..............................V....................................................................
SA7504	.............................C....................................................................................................
SA0763
SA5753
SA8795
B5505
B5631
B6538


	_________________________________________________________________
	         *       800       810       820       830       840
Cons. A1	YVPSALNPADDPSRGRLGLYRPLLRLPYRPTTGRTSLYAVSPSVPSHLPVRVHFASPLHVAWRPP
SA018	.................................................................
SA0127	.................................................................
SA197	.................................................................
SA2882	.......................................D.........................
SA4546	.................................................................
SA5623	.................................................................
SA5699	.................................................................
SA6513	.................................................................
SA7504	.................................................................
SA0763
SA5753
SA8795
B5505
B5631
B6538


	                                                       Terminal protein domain
	__________________________________________________________________________________________________________________________________
	        10        20        30        40        50        60    *   70        80        90       100       110       120       130
Cons. A2	MPLSYQHFRKLLLLDDGTEAGPLEEELPRLADADLNRRVAEDLNLGNLNVSIPWTHKVGNFTGLYSSTVPIFNPEWQTPSFPKIHLQEDIINRCQQFVGPLTVNEKRRLKLIMPARFYPTHTKYLPLDKG
B5168	...........................................................S........A..................................................N..........
B5175	................................V.................................................................................................
B5558	...................V.............................---------------------------------------------------------------------------------
B5654	...............SDNDT...................................................................................................K..........
B5672	.........R.....L..................................N...........................................E...................................
SA8857	..................................................................................................................................
SA9349	..................................................................................................................................
B6536	....HS............V..............F.............P..D...................V...............H...........................................
B6027
B7086
B7227


									                             Spacer domain
	__________________________________________________________________________________________________________________________________
	       140       150       160       170       180       190       200       210       220       230       240       250       260
Cons. A2	IKPYYPDQVVNHYFQTRHYLHTLWKAGILYKRETTRSASFCGSPYSWEQELQHGRLVIKTSQRHGDESFCSQPSGILSRSSVGPCIRSQLKQSRLGLQPHQGPLASSQPGRSGSIRARAHPSTRRYFGVE
B5168	......EH..................................................H...............................................R...........V.....QH....
B5175	......................................................................................................................I......C....
B5558	-------------------------------------------..............................A.......................K....S...............V......C....
B5654	.................................................D.P......N.........L.T...................Q........N........S.........T...........
B5672	.......H......................................................................................................G.......V......C....
SA8857	..................................................................................................................................
SA9349	..................................................................................................................................
B6536	...................................................P......Q.........................R.............................................
B6027	                                                                  ...R......................T............R..S......W..V......CS...
B7086	                                                                  ...R......................T............R..S......W..V......CS...
B7227	                                                                  ...........P....................................................


	__________________________________________________________________________________________________________________________________
	       270       280       290       300       310       320       330       340       350       360       370       380       390
Cons. A2	PSGSGHIDHSVNNSSSCLHQSAVRKAAYSHLSTSKRQSSSGHAVEFHCLPPSSAGSQSQGSVFSCWWLQFRNSKPCSEYCLSHLVNLREDWGPCDEHGEHHIRIPRTPARVTGGVFLVDKNPHNTAESRL
B5168	....R.......S.....Y......E....F................S......R...........................................................................
B5175	..................................................................................................................................
B5558	..................Y...........F.....H...........F.....R...........................................................................
B5654	..............................F.....K.........................................H...............N...................................
B5672	...........................H......................................................................................................
SA8857	..................................................................................................................................
SA9349	..................................................................................................................................
B6536	........Y.........N...........I.A.E..................T.............................I.........................................T....
B6027	........YRASS.................F.......P....M...SF.....R.....PI.........................L......TD..................................
B7086	........YRASK.................F.......P........SF.....R.....PI.........................L......TD..................................
B7227	..............................H..F......C.............E...........................................................................

								Reverse Transcriptase (RT) Domain
	__________________________________________________________________________________________________________________________________
	       400       410       420       430*      440       450       460       470       480       490       500       510       520
Cons. A2	VVDFSQFSRGITRVSWPKFAVPNLQSLTNLLSSNLSWLSLDVSAAFYHIPLHPAAMPHLLIGSSGLSRYVARLSSNSRINNNQYGTMQNLHDSCSRQLYVSLMLLYKTYGWKLHLYSHPIVLGFRKIPMG
B5168	..................................................................................................................................
B5175	..........V.......................................................................................................................
B5558	..................................................................................................................................
B5654	............................................................V..........H......................................R...................
B5672	..................................................................................K...............................................
SA8857	..................................................................................................................................
SA9349	..................................................................................................................................
B6536	..........V..........................................................................A............................................
B6027	..........V....................................................................H...H.AL.......................R.........IM........
B7086	..........A.....................T..............................................H...H.AL.......................R.........IM........
B7227	..........T....................................................................H..................................................


	__________________________________________________________________________________________________________________________________
	       530       540       550  **   560       570       580       590       600       610       620       630       640       650
Cons. A2	VGLSPFLLAQFTSAICSVVRRAFPHCLAFSYMDDVVLGAKSVQHREALYTAVTNFLLSLGIHLNPNKTKRWGYSLNFMGYIIGSWGTLPQDHIVQKIKHCFRKLPVNRPIDWKVCQRIVGLLGFAAPFTQ
B5168	..............................................S.................................V.................................................
B5175	..................................................................................................................................
B5558	........................................T.....S.................................V.................N...............................
B5654	..................................................................................................................................
B5672	............................................L.S.................................V............I....................................
SA8857	.................M............................S...................................................................................
SA9349	.................M............................S...................................................................................
B6536	..............V...............................S.....................................................................
B6027	.........................................I..L..............................H..........S.............................
B7086	.........................................I..L.S............................H..........S......................V......
B7227	..............................................S...................................................N.................


												RNase H Domain
	__________________________________________________________________________________________________________________________________
	       660       670       680       690       700 *     710       720       730*      740         *       760       770       780
Cons. A2	CGYPALMPLYACIQAKQAFTFSPTYKAFLSKQYMNLYPVARQRPGLCQVFADATPTGWGLAIGHQRMRGTFVAPLPIHTAELLAACFARSRSGAKLIGTDNSVVLSRKYTSFPWLLGCTANWILRGTSFV
B5168	..................................................................................................................................
B5175	..................................................................................................................................
B5558	..................................................................................................................................
B5654	......................................................................................................................A...........
B5672	.............................RQ...................................................................................................
SA8857	..................................................................................................................................
SA9349	..................................................................................................................................
B6536
B6027
B7086
B7227

	_________________________________________________________________
	         *       800       810       820       830       840
Cons. A2	YVPSALNPADDPSRGRLGLSRPLLRLPFQPTTGRTSLYAVSPSVPSHLPVRVHFASPLHVAWRPP
B5168	...................C.............................................
B5175	.................................................................
B5558	............................R....................................
B5654	...................Y.......................................I.....
B5672	...................Y.............................................
SA8857	.................................................................
SA9349	.................................................................
B6536
B6027
B7086
B7227


	                                                     Spacer Domain
	__________________________________________________________________________________________________________________________________
	       200       210       220       230       240       250       260       270       280       290       300       310       320
Cons. B	RHGDKSFCPQSSGILSRSPVGPCIQSQLRQSRLGPQPTQGQLAGLQQGGSGSIRAGIHSTPWGTVGVEPSSSGHTHNCASSSSSCLHQSAVRKAAYSPVSTSKRHSSSGHAVELHHVPPNSSRSQSQGSV
B5540	...........P...P..S..........K.......A......R..........RV.PS..........G..P.......................LI....G............F...........P.
B7081	...........T...P..S........F.K......SI......C..........RV.PS...F......G..........T...............LI....G............F.S.....K.....


										Reverse Transcriptase (RT) Domain
	__________________________________________________________________________________________________________________________________
	       330       340       350       360       370       380       390       400       410       420        *        440       450
Cons. B	LSCWWLQFRNSEPCSEHCLFHIVNLIEDWGPCTEHGEHRIRTPRTPARVTGGVFLVDKNPHNTTESRLVVDFSQFSRGNTRVSWPKFAVPNLQSLTNLLSSDLSWLSLDVSAAFYHLPLHPAAMPHLLVG
B5540	...........Q....Y..C.................................................................................N............................
B7081	..........R.....Y..C.....L...........................................................................N............................


	__________________________________________________________________________________________________________________________________
	       460       470       480       490       500       510       520       530       540       550**     560       570       580
Cons. B	SSGLSRYVARLSSNSRIINHQHRTMQNLHDSCSRNLYVSLMLLYKTYGRKLHLYSHPIILGFRKIPMGVGLSPFLLAQFTSAICSVVRRAFPHCLAFSYMDDVVLGAKSVQHLESLYAAVTNFLLSLGIH
B5540	...................N...A.....N....................................................................................T...............
B7081	......................GI..............................................................................M...........................


	_________________________________________________________
	       590       600       610       620       630
Cons. B	LNPHKTKRWGYSLNFMGYVIGSWGTLPQEHIVLKIKQCFRKLPVNRPIDWKVCQRIV
B5540	................................Q...M....................
B7081	........................S.......Q...L....................


									Terminal protein domain
	____________________________________________________________________________________________________________________________________
	        10          20        30        40        50        60  *     70        80        90       100       110       120       130
Cons. C	MPLSYQHFRKLLLLDD--EAGPLEEELPRLADEGLNRRVAEDLNLGNLNVSIPWTHKVGNFTGLYSSTVPVFNPDWQTPSFPHIHLKEDIINRCQQYVGPLTVNEKRRLKLIMPARFYPNLTKYLPLDKGIK
B5129	...............HGTA.................H.....................................E.......D....................................T............


												Spacer Domain
	__________________________________________________________________________________________________________________________________
	       140       150       160       170       180       190       200       210       220       230       240       250       260
Cons. C	PYYPEHTVNHYFQTRHYLHTLWKAGILYKRETTRSASFCGSPYSWEQELQHGRLVFQTSTRHGDEPFCSQSSGILSRSPVGPSVRSQFKQSRLGLQPQQGSMASGKPGRSGIIRARVHSTTRQSFGVEPS
B5129	......A.....K..............................................E.....S................C....L......P........R.NS....G......P...R.......


	__________________________________________________________________________________________________________________________________
	       270       280       290       300       310       320       330       340       350       360       370       380       390
Cons. C	GSGHIDNSASSASSCLHQSAVRKTAYSHLSTSKRQSSSGHAVELQHIPPSSARSQSEGPILSCWWLKFRNSKPCSDYCLSHIVNLLEDWGPCTEYGEHHIRIPRTPARVTGGVFLVDKNPHNTTESRLVV
B5129	.........NI....................A.....A...M.FHN....-----------.....Q...........................H...N...................S...........


								Reverse Transcriptase (RT) Domain
	__________________________________________________________________________________________________________________________________
	       400       410       420        *        440       450       460       470       480       490       500       510       520
Cons. C	DFSQFSRGSTHVSWPKFAVPNLQSLTNLLSSNLSWLSLDVSAAFYHLPLHPAAMPHLLVGSSGLPRYVARLSSTSRNINYQHGTMQDLHDSCSRNLYVSLLLLYKTFGRKLHLYSHPIILGFRKIPMGVG
B5129	..........K...................................................................................H.....M...Q.........................


	__________________________________________________________________________________________________________________________________
	       530       540       550**     560       570       580       590       600       610       620       630       640       650
Cons. C	LSPFLLAQFTSAICSVVRRAFPHCLAFSYMDDVVLGAKSVQHLESLFTSITNFLLSLGIHLNPNKTKRWGYSLNFMGYVIGSWGTLPQEHIVQKLKQCFRKLPINRPIDWKVCQRIVGLLGFAAPFTQCG
B5129	..............................................Y............................................I..I.H......V..........................


												RNase H Domain
	__________________________________________________________________________________________________________________________________
	       660       670       680       690         *       710       720        *        740       *         760       770       780
Cons. C	YPALMPLYACIQAKQAFTFSPTYKAFLCKQYLNLYPVARQRSGLCQVFADATPTGWGLAIGHQRMRGTFVAPLPIHTAELLAACFARSRSGAKLIGTDNSVVLSRKYTSFPWLLGCAANWILRGTSFVYV
B5129	....................A...V.........................................................................................................


	_______________________________________________________________
	       *         800       810       820       830       840
Cons. C	PSALNPADDPSRGRLGLYRPLLRLPFRPTTGRTSLYAVSPSVPSHLPDRVHFASPLHVAWRPP
B5129	................I..............................G....V.......K..


	                                                      Terminal protein domain
	__________________________________________________________________________________________________________________________________
	        10        20        30        40        50        60  *     70        80        90       100       110       120       130
Cons. D	MPLSYQHFRRLLLLDDEAGPLEEELPRLADEGLNRRVAEDLNLGNLNVSIPWTHKVGNFTGLYSSTVPVFNPHWKTPSFPNIHLHQDIIKKCEQFVGPLTVNEKRRLQLIMPARFYPNVTKYLPLDKGIK
B5503	...............E...........................................................................................K..........F...........
B5545	.....................................................................................................................K............
B6052	............................................D...............................................................................S.....
B7169	...............................D.............P.......................................................................K............
B7181	.........SV....E.................................................S........................R.........I......K..........F...........
PO1125	...............................D....................................................R................................K............
PO2896	..........-....N...............D....................................................R................................K............
B5594	.........K......D..............D..................................I...............................................................
B6078
B6612
B7197
PO1692
SA5256
CR417082


	                                                                               Spacer Domain
	__________________________________________________________________________________________________________________________________
	       140       150       160       170       180       190       200       210       220       230       240       250       260
Cons. D	PYYPEHLVNHYFQTRHYLHTLWKAGILYKRETTHSASFCGSPYSWEQELQHGAESFHQQSSGILSRPPVGSSLQSKHRKSRLGLQSQQGHLARRQQGRSWSIRAGIHPTARRPFGVEPSGSGHTTNLASK
B5503	...............................S...............................F.............H.........K.P...............V.................N......
B5545	...............................................D.........................................................F....................F...
B6052	................................................................................................................................N.
B7169	.................................R...........................................H.............................................N......
B7181	.............................................................................QQ...................G......V.........L.......N......
PO1125	.............................................................................S.............................................N......
PO2896	.............................................................................S.............................................N......
B5594	.................................R..........................................................K..............................N..H...
B6078	                                                     ....................................................F....................F...
B6612	                                                     ..........F.............H...........P...............V.......S.........N......
B7197	                                                     .......................................................................S...N.
PO1692	                                                     ........................S...........................F....................F...
SA5256	                                                     ....................................................F....................F...
CR417082	                                                     ..V.................................................V....................F.N.


	___________________________________________________________________________________________________________________________________
	        270       280       290       300       310       320       330       340       350       360       370       380       390
Cons. D	SASCLYQSP-VRKAAYPSVSTFEKHSSSGHAVELHNLPPNSARSQSERPVFPCWWLQFRNSKPCSDYCLSHIVNLLEDWGPCAEHGEHHIRIPRTPARVTGGVFLVDKNPHNTAESRLVVDFSQFSRGNYR
B5503	.....N...-AQ.....A...S.R........................SL.........D.......................................................................
B5545	.....HH..-.......A....A..........F.........................S..........L....................T....S................................H.
B6052	.....D...-..T....A................................................................T................................................
B7169	....I....-.......A........................G..G.G...................................................................................
B7181	.....H...-.......A...............................................................................................................H.
PO1125	........TVIK...N.A.....R..............Q......G....................................I................................................
PO2896	........S-.......A................................................................T................................................
B5594	....I....-D......A.....R............F........G...................................................................................H.
B6078	.....H...-.......A...............F....................................L.........................S..................................
B6612	.....N...-.K.....A...S..........................SL.........D........I..............................................................
B7197	........S-.....D.A.................................................................................................................
PO1692	.....H...-D......A...............F...S.....................S..........L.........................S..................................
SA5256	.....H...-.....N.A...............F....................................L.........................S..................................
CR417082	.T..IH...-.....N.A.....R............F........................N........L...........T.............S..................................


	                                                     Reverse Transcriptase (RT) Domain
	____________________________________________________________________________________________________________________________________
	       400       410       *         430       440       450       460       470       480       490       500       510       520
Cons. D	VSWPKFAVPNLQSLTNLLSSNLSWLSLDVSAAFYHLPLHPAAMPHLLVGSSGLSRYVARLSSNSRIFNHQHGTMQNLHDSCSRNLYVSLLLLYQTFGRKLHLYSHPIILGFRKIPMGVGLSPFLLAQFTS
B5503	...................................I.........................................................K...W................................
B5545	..................................................................L.......S....C..K...............................................
B6052	..................................................................I.Y.............................................................
B7169	...................................I..................................R........Y..................................................
B7181	...................................I.........................................................K...W................................
PO1125	......................................................................R........D..................................................
PO2896	......................................................................R........Y..................................................
B5594	......................................................................R........Y..................................................
B6078	..................................................................L.N.....PD...Y..................................................
B6612	.............................................................................................K...W................................
B7197	....................................................................Y.......M.....................................................
PO1692	..................................................................L.......P....Y..................................................
SA5256	...........................................................V......L.......P.......................................................
CR417082	..................................................................L.......P.......................................................


	__________________________________________________________________________________________________________________________________
	       530         **      550       560       570       580       590       600       610       620       630       640       650
Cons. D	AICSVVRRAFPHCLAFSYMDDVVLGAKSVQHLESLFTAVTNFLLSLGIHLNPNKTKRWGYSLHFMGYVIGCYGSLPQDHIIQKIKECFRKLPVNRPIDWKVCQRIVGLLGFAAPFTQCGYPALMPLYACI
B5503	.............................S.....................T...................W.........H................................................
B5545	..............................................................N..............E....................................................
B6052	..................................................................................................................................
B7169	...........................T........................Y.........N...................................................................
B7181	....................................................Y..................W........RH................................................
PO1125	....................................................D.........N...................................................................
PO2896	..............................................................N...................................................................
B5594	..............................................................N.................V....................
B6078	..............................................................N..............E..............I........
B6612	...........................T.......................T...Q...............W.........H...................
B7197	......................................................................S......E.......................
PO1692	..............................................................N.......S......E..............I........
SA5256	....................................................S.........N..............E..V...........I........
CR417082	.............................


											RNase H Domain
	__________________________________________________________________________________________________________________________________
	       660       670       680        *        700       710       *         730      *740       750       760       770      *780
Cons. D	QSKQAFTFSPTYKAFLCKQYLNLYPVARQRPGLCQVFADATPTGWGLVMGHQRMRGTFLAPLPIHTAELLAACFARSRSGANILGTDNSVVLSRKYTSFPWLLGCAANWILRGTSFVYVPSALNPADDPS
B5503	...............................................AI.........Q.......................................................................
B5545	..................................................L................................I..............................................
B6052	.A........................................................P.......................................Y...............................
B7169	..R...............................................................................................................................
B7181	...............................................AI.........Q.......................VI..............................................
PO1125	..R...............................................................................................................................
PO2896	..R.......................................................P.......................................................................
B5594
B6078
B6612
B7197
PO1692
SA5256
CR417082


	____________________________________________________
	       790       800       810       820       830
Cons. D	RGRLGLSRPLLRLPFRPTTGRTSLYADSPSVPSHLPDRVHFASPLHVAWRPP
B5503	.....IY....C........................................
B5545	....................................................
B6052	....................................................
B7169	....................................................
B7181	.....I.....C........................V...............
PO1125	....................................................
PO2896	...............Q....................V...............
B5594
B6078
B6612
B7197
PO1692
SA5256
CR417082


									Terminal protein domain
	__________________________________________________________________________________________________________________________________
	        10        20        30        40        50        60  *     70        80        90       100       110       120       130
Cons. E	MPLSYQHFRRILLLDEEAGPLEEELPRLADEDLNRRVAEDLNLQLPNVSIPWTHKVGNFTGLYSSTIPVFNPNWKTPSFPDIHLHQDIINKCEQFVGPLTVNEKRRLNLVMPARFFPISTKYLPLEKGIK
B6156	.........K......................F..........R......................................................................................
B6197	......Y...L.....G.................................................................................................................
B7047	..........L..................................................................................................I....................
B7052	..................................................................V...............................................................
B7102	..................................................................................................................................
B7112	.........S....................D...................................................................................................
B6507
B6662
B7152


												Spacer Domain
	________________________________________________________________________________________________________________________
	       140       150       160       170       180       190       200       210       220       230       240       250
Cons. E	PYYPDNVVNHYFQTRHYLHTLWKAGILYKRETTRSASFCGSPYSWEQELHHGAFLDGPSRMGEESFHHQSSGIFSRPPVGSSIQSKHQKSRLGPQSQQRPLDRSQQGRSGSIRAGVHSPT
B6156	..............................................HD...............K........................Q.....K........R................
B6197	...............................S............................V.K..V......................................................
B7047	...............................SA.................................................................................WG....
B7052	.................................................................................................K....G..........V......
B7102	................................................................................................................W.R.....
B7112	.....................................................L.........K.........L............................G........L........
B6507	                                                                ......................Y.......K......E.............A....
B6662	                                                                ..................................................R.....
B7152	                                                                ......................Y...............TK................


	__________________________________________________________________________________________________________________________________
	       260       270       280       290       300       310       320       330       340       350       360       370       380
Cons. E	RRPFGVEPSGSRHAKNIASRSASCLHQSAVRKAAYPNHSTFERHSSSGHAVEFHNIPPSSAGSQSKRPVFSCWWLQFRNSEPCSDYCLTHLVNLLEDWGPCTEHGKHHIRIPRTPARVTGGVFLVDKNPH
B6156	................................................R.....K......E........T..................................R........................
B6197	.....M.......T.....K.............................T...........E........T...........................................................
B7047	......................................................K..................................................N........................
B7052	..................................................................................................................................
B7102	.........................................................................................................E........................
B7112	..................................................................................................................................
B6507	.................................................................T...S............................................................
B6662	..................N............................................................K..................................................
B7152	.....M.......................................................E.----.ASY.....................T.....................................


									Reverse Transcriptase (RT) Domain
	__________________________________________________________________________________________________________________________________
	       390       400       410       420       *         440       450       460       470       480       490       500       510
Cons. E	NTAESRLVVDFSQFSRGSSRVSWPKFAVPNLQSLTNLLSSNLSWLSLDVSAAFYHLPLHPAAMPHLLVGSSGLSRYVARLSSNSRIINHQYGTLPNLHDSCSRNLYVSLMLLFKTFGRKLHLYSHPIIMG
B6156	..................................................................................................................................
B6197	..T.....................................D.........................................................................................
B7047	...................................................................................................Y............................L.
B7052	..................................................................................................................................
B7102	..................................................................................................................................
B7112	.......................................T..........................................................................................
B6507	...............................................................................................D..........D.......................
B6662	..................................................................................................................................
B7152	..............................................................................................S...................................


	__________________________________________________________________________________________________________________________________
	       520       530       540         **      560       570       580       590       600       610       620       630       640
Cons. E	FRKIPMGVGLSPFLLAQFTSAICSVVRRAFPHCLAFSYMDDVVLGAKSVQHLESLYTSVTNFLLSLGIHLNPNKTKRWGYSLNFMGYVIGSWGSLPQEHIRMKIKDCFRKLPVNRPIDWKVCQRIVGLLG
B6156	.................................................................................................D...L............................
B6197	.....................L................................I...................................G..........L............................
B7047	..................................................................................................................................
B7052	..................................................................................................................Q...............
B7102	..................................................................................H..................L............................
B7112	..................................................................................................................................
B6507	...................................................................................................
B6662	..........................................................I.............H..Q.......................
B7152	........................................................................H..........................


												RNase H Domain
	__________________________________________________________________________________________________________________________________
	       650       660       670       680       690        *        710       720       *         740      *750       760       770
Cons. E	FAAPFTQCGYPALMPLYACIQSKQAFTFSPTYKAFLCKQYLNLYPVARQRPGLCQVFADATPTGWGLAIGHQRMRGTFVAPLPIHTAELLAACFARSRSGAKLIGTDNSVVLSRKYTSFPWLLGCAANWI
B6156	.....................................Q..M....................................................................................T....
B6197	..............................I......N..............................M.............................................................
B7047	..................................................................................................................................
B7052	.........................................T........................................................................................
B7102	........................................M.........................................................................................
B7112	.....................................................................................................N............................
B6507
B6662
B7152


	________________________________________________________________________
	       780      *790       800       810       820       830       840
Cons. E	LRGTSFVYVPSALNPADDPSRGRLGIYRPLLRLPFQPTTGRTSLYAVSPSVPSHLPDRVHFASPLHVAWRPP
B6156	.........................V........Y..S..................................
B6197	..........................S.............................V...............
B7047	...................................R....................................
B7052	.........................VC.............................................
B7102	.........................V..............................................
B7112	..........................S.............................................
B6507
B6662
B7152
```

**SUPPLEMENTARY FIGURE S6. Alignment of Pol amino acid sequences.** Sequences from HBV-infected blood donors with HBsAg levels >100 IU/mL and either ID-NAT nonreactive or reactive results with HBV DNA <20 IU/mL were aligned against genotype-matched consensus sequences obtained from HBsAg+/HBV DNA+ donor strains. Amino acids identical to the reference consensus are indicated by dots and deletions by dashes. Functional domains within the Pol protein are represented by distinct colors. Critical motifs are highlighted in red, and essential functional residues are marked with an asterisk.

**SUPPLEMENTARY TABLE S3.** Average Pol-domain amino acid diversity in cases and controls stratified by HBV genotype.

| **Pol domains** |  | **HBV_A1_** | | |  | **HBV_A2_** | | |  | **HBV_D_** | | |  | **HBV_E_** | | |
| --- | --- | --- | --- | --- | --- | --- | --- | --- | --- | --- | --- | --- | --- | --- | --- | --- |
|  |  | **Cases**  **(N=9)** | **Controls**  **(N=16)** | ***p*** |  | **Cases**  **(N=7)** | **Controls**  **(N=16)** | ***p*** |  | **Cases**  **(N=7)** | **Controls**  **(N=19)** | ***p*** |  | **Cases**  **(N=6)** | **Controls**  **(N=27)** | ***p*** |
| **Terminal protein** |  | 0.8  (0.0-2.2) | 0.8  (0.0-2.7) | ns |  | 2.7  (0.0-4.9) | 1.1  (0.0-4.4) | ns |  | 2.7  (0.0-4.9) | 1.7  (0.0-4.4) | ns |  | 1.7  (0.0-2.7) | 0.6  (0.0-1.1) | <0.05 |
| **Spacer** |  | 3.3  (3.3-5.3) | 3.3  (1.8-5.3) | ns |  | 2.4  (0.0-7.3) | 1.2  (0.6-3.6) | ns |  | 5.2  (2.6-9.7) | 5.2  (1.3-7.8) | ns |  | 2.4  (1.2-5.5) | 1.2  (0.0-2.4) | <0.05 |
| **RT** |  | 1.4  (0.8-2.8) | 1.2  (0.9-2.1) | ns |  | 0.5  (0.3-1.9) | 0.6  (0.0-1.8) | ns |  | 1.9  (1.1-1.8) | 2.1  (1.1-6.3) | ns |  | 0.9  (0.3-2.2) | 0.6  (0.0-1.5) | ns |
| **RNase H** |  | 0.7  (0.0-0.7) | 0.6  (0.0-1.8) | ns |  | 0.7  (0.0-2.1) | 0.6  (0.0-1.9) | ns |  | 1.4  (0.0-5.7) | 1.2  (0.0-3.7) | ns |  | 1.4  (0.7-2.9) | 1.2  (0.0-2.5) | ns |

**SUPPLEMENTARY FIGURE S7.** HBV RNA semi-quantification in plasmas of 37 case and 25 control samples. For clarity, individuals with negative RNA results (33 cases and 9 controls) are excluded from the figure. AU, arbitrary unit.
